# Invasive cane toads are unique in shape but overlap in ecological niche compared to Australian native frogs

**DOI:** 10.1002/ece3.3253

**Published:** 2017-08-17

**Authors:** Marta Vidal‐García, J. Scott Keogh

**Affiliations:** ^1^ Ecology and Evolution Research School of Biology The Australian National University Canberra ACT Australia

**Keywords:** amphibian, biological invasions, body shape, empty niche, morphology, niche breadth

## Abstract

Invasive species are an important issue worldwide but predicting invasiveness, and the underlying mechanisms that cause it, is difficult. There are several primary hypotheses to explain invasion success. Two main hypothesis based on niche spaces stand out as alternative, although not exclusive. The empty niche hypothesis states that invaders occupy a vacant niche space in the recipient community, and the niche competition hypothesis states that invaders overlap with native species in niche space. Studies on trait similarity/dissimilarity between the invader and native species can provide information on their niche overlap. Here, we use the highly invasive and well‐studied cane toad (*Rhinella marina*) to test these two hypotheses in Australia, and assess its degree of overlap with native species in several niche dimensions. We compare extensive morphological and environmental data of this successful invader to 235 species (97%) of native Australian frogs. Our study is the first to document the significant morphological differences between the invasive cane toad and a continent‐wide frog radiation: despite significant environmental overlap, cane toads were distinct in body size and shape from most Australian frog species, suggesting that in addition to their previously documented phenotypic plasticity and wide environmental and trophic niche breadth, their unique shape also may have contributed to their success as an invasive species in Australia. Thus, the invasive success of cane toads in Australia may be explained through them successfully colonizing an empty niche among Australian anurans. Our results support that the cane toad's distinct morphology may have played a unique role in the invasiveness of this species in Australia, which coupled with a broad environmental niche breadth, would have boosted their ability to expand their distribution across Australia. We also propose RLLR (Relative limb length ratio) as a potentially useful measure of identifying morphological niche uniqueness and a potential measure of invasiveness potential in anuran amphibians.

## 1 INTRODUCTION

Human‐mediated introduction of non‐native species to new habitats has occurred for thousands of years (di Castri, Hansen, & Debussche, 1990). Most of the time the introduction fails, but occasionally a species will establish and become invasive. The impact of these invasive alien species on native species assemblages and habitats has become a key ecological problem (Bergmans & Blom, 2001; Colautti & Barrett, 2013; Simberloff, Parker, & Windle, 2005), and improving our understanding of the drivers of successful invasion is paramount (Hayes & Barry, 2008). The attributes of invaders have received a lot of attention, in particular their behavioral and personality traits (Chapple et al., genetic variation (Tsutsui, Suarez, Holway, & Case, 2000; Lee, 2002), physiological tolerance (Zerebecki & Sorte, 2011), and dispersal capacity (Václavík & Meentemeyer, 2009). Studies on these traits, when coupled with information on ecological impact and interactions with native fauna and flora (Didham, Tylianakis, Gemmell, Rand, & Ewers, 2007; Shine, 2014; Vilà et al., 2011), shed insight into the mechanisms underpinning invasion success (Chapple, Simmonds, & Wong, 2012), which improves our ability to plan effective mitigation strategies (Kolar & Lodge, 2001; Van Kleunen, Dawson, Schlaepfer, Jeschke, & Fischer, 2010).

Community ecology theory provides a conceptual framework to assess the factors that might promote successful invasiveness based on niche opportunities (Shea & Chesson, 2002; Simberloff, 1995). Species’ niches are defined by the whole range of environmental conditions, including all their biotic and abiotic interactions with the ecosystem, within which they can thrive (Hutchinson, 1957). Determining the invader's niche breadth and its niche overlap with native species may be highly informative as invaders could limit the distribution of species in the native community (Ricklefs, 1987; Ricklefs & Miles, 1994). There are two main hypotheses based on niche spaces to explain how invasive species can establish: The empty niche hypothesis predicts that invasive species are more successful when they occupy a portion of available niche space that the native community does not utilize (MacDougall, Gilbert, & Levine, 2009; Stachowicz & Tilman, 2005). Under this hypothesis, the invader exhibits traits that are well suited to the ecological conditions of the new environment, but that do not overlap with native species (Azzurro et al., 2014). The niche competition or competitive exclusion hypothesis predicts that if two species that occur together also share the same niche, one species will be eliminated or displaced, because complete competitors cannot coexist (Bøhn, Amundsen, & Sparrow, 2008; Hardin, 1960). Invaders that are more efficient than natives at exploiting a shared resource will negatively impact native species and displace them from their original niche (Azzurro et al., 2014; Bøhn et al., 2008; Duncan & Williams, 2002). These two hypotheses, while strictly nonexclusive, could potentially be alternative to one another.

Discriminating between both hypotheses requires a detailed understanding of the ecology and phenotype of the invading species, as well as the available niches and the ways in which native species are adapted to fill those niches. As phenotypic traits greatly influence the environmental range of a species, their distribution in ecological space is also often correlated with distribution in morphological space (Ricklefs & Miles, 1994). Thus, morphological traits could be used as a proxy for a species’ ecological niche in a community, especially when those morphological traits are correlated with functional traits, such as performance capacity (Azzurro et al., 2014; Ricklefs & Miles, 1994). A number of morphological traits have been used previously in several taxa as a way to determine niche overlap among species (Gatz, 1979; Losos, 1990). As morphological plasticity broadens the range of environmental conditions under which a species could thrive, understanding the body size and shape patterns of a species and their plasticity would capture its niche breadth (Whitlock, 1996). In invasive species biology, a great deal of research attention is devoted to studying the ecology of invasive species in new habitats, and the impact of establishment on native species, but comparatively little attention is given to directly quantifying niche position and breadth for both invaders and natives. Here we exploit one of the best‐known biological invaders to discriminate between the two competing hypotheses of empty niche and niche competition.

The highly invasive cane toad, *Rhinella marina,* is native to Central and tropical South America (Zug & Zug, 1979), but was introduced across the globe, including Australia, and has successfully invaded more than twenty countries to date (Lever, 2001). The cane toad is one of the World's worst alien invasive species (Lowe, Browne, Boudjelas, & De Poorter, 2000) and its impact on native fauna has been studied extensively (Letnic, Webb, & Shine, 2008; Shine, 2010, 2014; van Winkel & Lane, 2012). It has been particularly well studied in Australia (a continent where no other members of the Family Bufonidae naturally occur; Anstis, 2013), where they were introduced in 1935 as part of an unsuccessful program to control cane beetles (Freeland & Martin, 1985). Cane toads are among the largest anuran species in the World (with snout–vent length of up to 380 mm, but usually around 150 mm; Lever, 2001) and are known to be extremely morphologically plastic, especially in their limb lengths (Phillips, Brown, Webb, & Shine, 2006).

Here we assess the morphological niche overlap between cane toads and Australian frog species in order to discriminate between the empty niche and competitive exclusion hypotheses. Under the empty niche hypothesis, we would expect cane toads to fill a unique morphological niche not occupied by Australian native frog species. Thus, cane toads are expected to be morphologically distinct from endemic Australian species, most likely also occupying a different environmental or trophic niche than native frogs. The competitive exclusion hypothesis predicts the invaders’ morphological niche would overlap with native species’ phenotypic traits. Under this scenario, cane toads are expected to be morphologically similar to Australian frogs and would likely overlap in trophic niche and habitat use. In order to evaluate and discriminate between these hypotheses, we measured and analyzed body size and shape of the cane toad in relation to each of the Australian frog species and compared limb length ratios between cane toads and each Australian frog clade. We also compared environmental niche position and breadth between the cane toad and endemic frog clades. We discuss the morphological niche of the cane toad in the context of their environmental niche, phylogenetic constraints, behavioral adaptations, and invasiveness success in Australia.

## 2 MATERIALS AND METHODS

### 2.1 Morphological traits

We collected detailed morphological data for 54 adult specimens of cane toad (*Rhinella marina*), and the selected specimens spanned the full invasion history in Australia (1930s to present day; Lever, 2001) and the entire current distribution in order to capture the whole range of phenotypic variation. We included both males and females to test the potentially confounding effects of sexual dimorphism in the comparison with the other Australian frog species. For each specimen, we collected 34 external linear measurements to the nearest 0.1 mm with digital callipers, from which we selected 25 variables for further analyses, following established methods (Vidal‐García, Byrne, Roberts, & Keogh, 2014). A list of the species and specimens used in this study is provided in the supplementary materials (Appendix S1). All measurements were taken by the same person (MVG) to ensure consistency in the data collection. We evaluated our cane toad data against equivalent data from two of our published studies on native Australian frog species that included 127 of the 131 species of myobatrachid frogs (Vidal‐García, Byrne, Roberts, & Keogh, 2014) and 84 of 86 species of hylid frogs (Vidal‐García & Keogh, 2015), but also against data from 23 of 24 species of microhylid frogs, and the only species of ranid frog. Together these studies included comprised morphological information for 1216 specimens, 45 genera or clades (hylid clades are previously described in Vidal‐García & Keogh, 2015), and 97.5% of all Australian frog species. As there was no evidence of sexual dimorphism in any shape variable within the cane toads, sex was not taken into account when comparing morphological data to Australian clades (see Results).

### 2.2 Environmental variables

Environmental data were obtained from The Atlas of Living Australia online database (Atlas of Living Australia) using distributional data of each frog species. We assembled 12 environmental variables relevant to frogs (as per Vidal‐García & Keogh, 2015): Annual mean evaporation, precipitation in the warmest quarter (Bio18), temperature in the warmest period (Bio05), soil nutrient status, annual mean moisture index, topographic slope, and mean net primary productivity, precipitation in the driest quarter (Bio17) seasonality of precipitation (Bio15), seasonality of radiation (Bio23), radiation in the warmest quarter (Bio26), and moisture on the highest quarter (Bio32). Each environmental record was gathered from geographical information for each specimen record based on 10 × 10 km blocks in the Universal Transverse Mercator coordinate system (UTM) for each species’ distribution. We also manually added all records from the Queensland Museum that currently are missing from The Atlas of Living Australia. In order to avoid a biased characterization of the environmental variables of the habitat occupied by each species, we used unduplicated 10 × 10 km UTM blocks, irrespective of the total number of specimen records in a single UTM. We assembled this information for every Australian frog species and the cane toad, resulting in 119,531 unduplicated records for each environmental variable.

### 2.3 Statistical analyses

We used principal components analysis (PCA) to reduce the dimensionality of the morphological data set for both the 25 raw variables. We then performed an analysis of variance (ANOVA) test on the following variables: SVL, the first three PC for the raw morphological PCA (PC 1raw, PC 2raw, PC 3raw), and the relative limb length ratio (RLLR: Arm length/leg length) among each of the Australian frog genera and clades. RLLR is particularly relevant because limb length has been extensively studied in cane toads (Phillips et al., 2006) and is known to be a good predictor of ecological niche in Australian frogs (Vidal‐García & Keogh, 2015; Vidal‐García, Byrne, Roberts, & Keogh, 2014). We also performed PCA and ANOVAs on the size‐corrected morphological data set (details on Appendix S2). We then performed post hoc pairwise comparisons on SVL, RLLR, and the two‐first size‐corrected PCs between each Australian clade and the cane toad using Dunnett's tests, in order to assess which clades were significantly different to the cane toad. We also calculated the SD of each variable as a measure of morphological variability within cane toads and compared them to those from each Australian frog clades with Bartlett's test and *F*‐test one‐way analysis of variance in order to test for homogeneity of variances and variance differences among groups. Additionally, we performed several Kruskal–Wallis tests (nonparametric analyses of variance by ranks) within cane toads to assess whether there were any morphological differences between males and females of *R. marina* for all the principal component variables, snout–vent length (SVL) and RLLR.

We also used PCA to reduce the dimensionality of the environmental data set. We performed ANOVAs and post hoc pairwise comparisons using Dunnett's tests as well as for the first two environmental PCs, in order to assess which clades were significantly different to the cane toad. These analyses depicted differences in niche position between Australian frog clades and the cane toad, based on environmental values from the whole geographic distribution of each species. We also used Bartlett's test and *F*‐test one‐way analysis of variance in order to test for variance differences among groups, as a proxy for environmental niche breadth differences.

### 2.4 Phylogenetic comparative analyses

In order to compare morphological niches among Australian frogs and cane toads in a phylogenetic context, we generated a phylogenetic hypothesis for Australian hylids, Australian microhylids, myobatrachids, *Rana daemeli,* and *Rhinella marina*, using *Xenopus muelleri* as an outgroup. Mitochondrial (12s and 16s) sequence data were obtained from Rosauer, Laffan, Crisp, Donnellan, and Cook (2009), CJ Hoskin et al. (in prep), JS Keogh, D Moore, PG Byrne, DJ Roberts (in prep), and Pyron (2014), in order to generate a Bayesian phylogenetic tree (Figure S1). Because our goal was not to infer a new phylogeny, we constrained our analyses to ensure the resultant topology did not differ from previously published phylogenetic analyses of the individual families. The phylogeny was highly consistent with Pyron's (2014) assessment of the phylogenetic history of the World's amphibians. We evaluated the magnitude of phylogenetic signal in multivariate data in the morphological and environmental variables using Blomberg's *K* statistic's generalization for multivariate data (*K*_mult_; Adams, 2014a) with *geomorph* (Adams & Otárola‐Castillo, 2013). We performed a phylogenetic ANOVA for both univariate and multivariate data in *geomorph* (Adams, 2014b), to test whether phylogeny affected morphological traits, environmental variables, and RLLR. We also performed phylogenetic regression models using this function to test the correlation between sets of traits. We then performed a phylogenetic PCA, on all morphological variables, with *phytools* (Revell, 2012). Phylogenetic ANOVAs and phylogenetic PCA were also ran on the size‐corrected data set (Appendix S2).

## 3 RESULTS

### 3.1 Morphological variation

**Figure 1:**
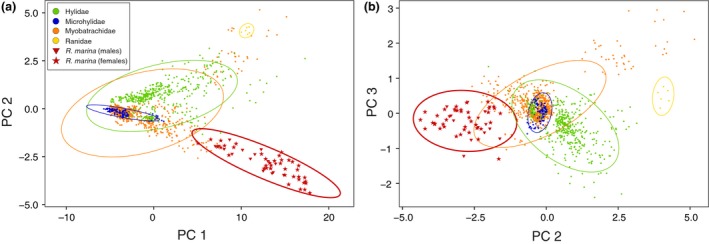
(a) Scatterplot of PC 1 and PC 2 values of the morphological data set showing the size and shape differences among Australian frog families and the cane toad *Rhinella marina*. (b) Scatterplot of PC 2 and PC 3 values of the morphological data set showing the shape differences among Australian frog families and *R. marina*. Both males and females of *R. marina* are depicted separately

**Figure 2:**
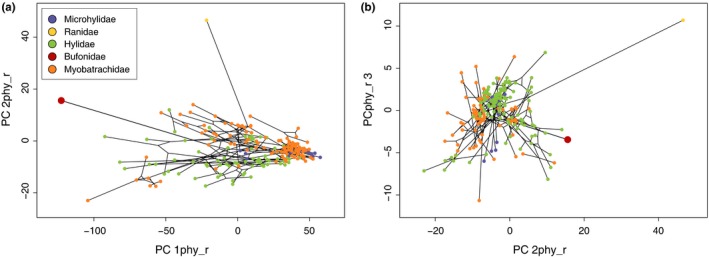
(a) Phylomorphospace of PC 1phy_r and PC 2phy_r values of the raw morphological data set showing the size and shape differences among Australian frog families and the bufonid *Rhinella marina*, using *phytools* (Revell, 2012). (b) Phylomorphospace of PC 2phy_r and PC 3phy_r values of the raw morphological data set showing the size and shape differences among Australian frog families and *R. marina*

The first principal component (PC 1raw) accounted for 88.76% of the total raw morphological variation across Australia's native frogs and the cane toad. As expected, PC 1 was highly correlated with snout–vent length (SVL) ($R_{\mathrm{adj}}^{2}=0.9458$, *p* < .0001; Figure 1a; Table S1). PC 2raw and PC 3raw represented 4.26% and 1.31% of shape variation, respectively. PC 2raw was most strongly correlated with the degree of toe webbing ($R_{\mathrm{adj}}^{2}=0.4034$, *p* < .0001) and toe length ($R_{\mathrm{adj}}^{2}=0.216$, *p* < .0001), whereas PC 3raw most strongly correlated with length of the snout (naris to snout length: $R_{\mathrm{adj}}^{2}=0.1064$, *p* < .0001; Figure 1b). Taking phylogenetic relationships into account, first principal component of the phylogenetic PCA (PCp 1phy_r) accounted for 96.01% of the total morphological variation across all frog species. PCp 2phy_r and PCp 3phy_r represented 2.06% and 0.53% of shape variation, respectively (Figure 2a,b). As in the non‐phylogenetic PCA, PCp 1phy_r was highly correlated with snout–vent length (SVL) ($R_{\mathrm{adj}}^{2}=0.9639$, *p* < .0001; Table S2). PCp 2phy_r's variance was mostly explained by (but not correlated to) degree of toe webbing ($R_{\mathrm{adj}}^{2}=0.186$, *p* < .0001), while PCp 3phy_r was correlated with shape of the snout (internarial length: $R_{\mathrm{adj}}^{2}=0.34$, *p* < .0001). Results for the size‐corrected and the phylogenetically size‐corrected data sets are summarized in the Appendix S2, Tables S3, S4, S5, and displayed in Figures S2 and S3AB. Body size (SVL) was the main predictor of morphological differences between cane toads and all the Australian frog species (*F*_1, 1214_ = 1069, *p* < .0001; Figure 1a). The ANOVA on body size (SVL) using genera and clades as a factor was also significant (*F*_45, 1170_ = 152.6, *p* < .0001). Post hoc comparisons of SVL between the cane toad and each clade of Australian native frog with Dunnett's test indicated that all Australian frog clades were significantly different to the cane toad (Table S5). ANOVA's results on raw body shape (PC 2raw and PC 3raw) also demonstrate that cane toads are different from most of the other clades (*F*_45, 1170_ = 244.9, *p* < .0001 for PC 2raw, and *F*_45, 1170_ = 112.1, *p* < .0001 for PC 3raw; *F*_1, 1214_ = 477, *p* < .0001 for PC 2raw, and *F*_1, 1214_ = 13.39, *p* = .0003 for PC 3raw; Figure 1b, Table S3).

**Figure 3:**
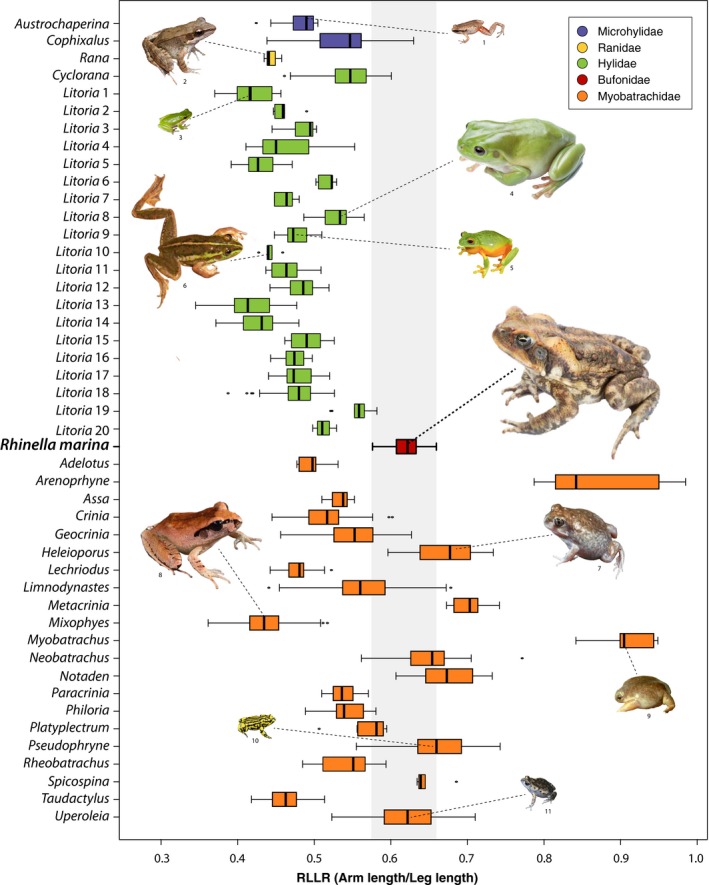
Boxplot of RLLR (Relative Limb Length Ratio: Arm length/Leg length) per clade. Morphological niche breadth of *Rhinella marina* is displayed for overlap comparisons with Australian frog clades. Representative species depicted: (1) *Austrochaperina gracilipes*, (2) *Rana daemeli*, (3) *Litoria fallax*, (4) *L. caerulea*, (5) *L. xanthomera*, (6) *L. dahlii*, (7) *Heleioporus eyrei*, (8) *Mixophyes carbinensis*, (9) *Myobatrachus gouldii*, (10) *Pseudophryne corroboree*, and (11) *Uperoleia laevigata*. Whiskers are defined by 1.5 times the interquartile range (IQR)

There were significant differences in relative limb length ratio (RLLR) between the cane toad and almost all other Australian frog clades (*F*_45, 1170_ = 173, *p* < .0001; *F*_1, 1214_ = 40.05, *p* < .0001; Figure 3), and this also was true following phylogenetic correction (*F*_45, 170_ = 1.658, *p* = .001; *F*_1, 214_ = 3.1927, *p* = .002). There was no overlap in RLLR between the cane toad and any hylid, microhylid, or ranid species. Similarly, there was no overlap between the cane toad and most of the myobatrachid genera. Only two myobatrachid frog genera showed some degree of overlap and did not significantly differ in Dunnett's test for RLLR: *Uperoleia* spp. and *Spicospina flammocaerulea* (Table S5). Bartlett's test, used to test for variance differences among groups, displayed morphological niche breadth differences among different clades (Table S6).

Morphological niche breadth was typically wider in cane toads than in native frog clades, especially in SVL, but there were no significant differences in higher variance in other variables (Table S7). Differences in SVL variance between cane toads and Australian clades were probably due to the way we sampled the cane toads (sampling on purpose species from different locations and capture years in order to capture their whole variation range, while the sampling was randomized for Australian frogs). Cane toads displayed sexual size dimorphism with females larger than males in both PC 1raw (Kruskal–Wallis $\chi_{1}^{2}=6.8476$, *p* = .009; Figure 1a) and SVL (*F*_1, 52_ = 5.326, *p* = .021); however, there was no evidence of sexual dimorphism in any shape variable (PC 2raw: Kruskal–Wallis $\chi_{1}^{2}=2.4169$, *p* = .12; PC 3raw: Kruskal–Wallis $\chi_{1}^{2}=0.0896$, *p* = .7647; PC 1sc: Kruskal–Wallis $\chi_{1}^{2}=1.5039$, *p* = .220; PC 2sc: Kruskal–Wallis $\chi_{1}^{2}=1.4106$, *p* = .235; PC 3sc: Kruskal–Wallis $\chi_{1}^{2}=1.5516$, *p* = .213; RLLR: *F*_1, 52_ = 2.5385, *p* = .111), so sex was not taken into account when comparing morphological data to Australian clades.

### 3.2 Environmental variation

**Figure 4:**
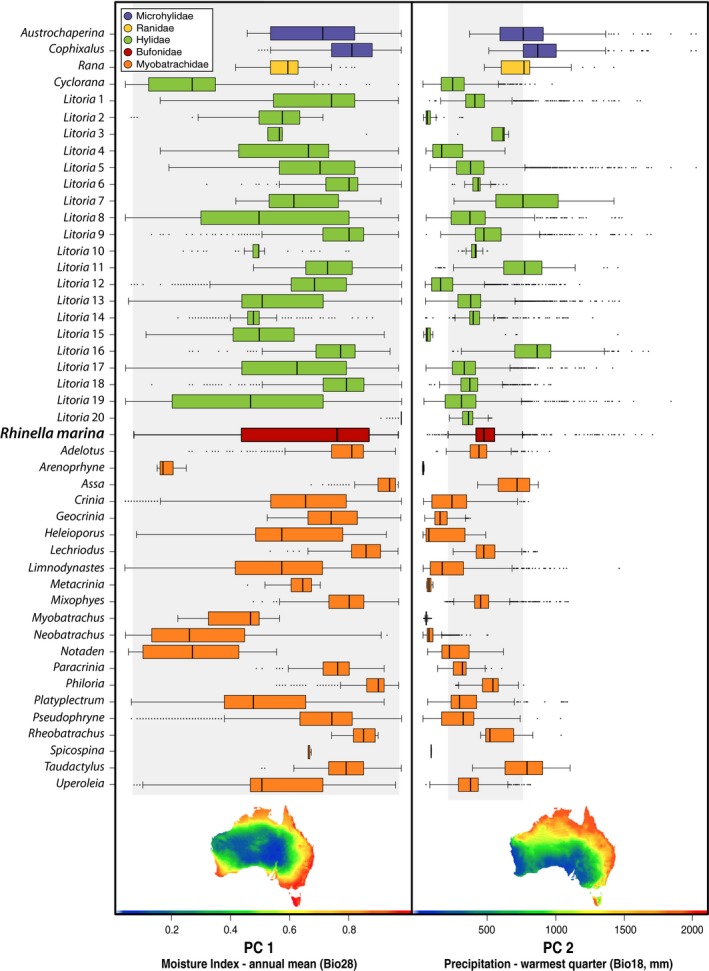
Boxplot of Moisture Index—annual mean (Bio28) based on the environmental PC 1 and Precipitation—warmest quarter (Bio18, mm) based on PC 2, per clade. Environmental niche breadth of *Rhinella marina* is displayed for overlap comparisons with Australian frog clades. Whiskers are defined by 1.5 times the interquartile range (IQR)

In the environmental dataset, the first two principal components explained 66.53% of the overall variation. PC 1 accounted for 44% of the environmental variability, PC 2 an additional 22.53%, and PC 3 an additional 10.06% (Table S8). PC 1 was correlated with habitat humidity (annual mean climatic moisture index: Bio28, $R_{\mathrm{adj}}^{2}=0.8768$, *p* < .0001), while PC 2 was correlated with precipitation in the warmest quarter (Bio18, $R_{\mathrm{adj}}^{2}=0.7455$, *p* < .0001) and seasonality of radiation (Bio23, $R_{\mathrm{adj}}^{2}=0.7151$, *p* < .0001), and PC 3 was correlated with soil nutrient status. Cane toads overlapped with Australian frogs in environmental niche for both PC values and environmental variables (Figure 4). Bartlett's test, used in order to test for variance differences among groups, was significant for all environmental variables, displaying strong differences of environmental niche breadth among different frog clades (Table S9). Dunnett's tests depicted differences in niche position from some clades (Table S5), but they still occupied a broad region in the center of the Australian frogs’ “ecospace” obtained with the PC values (Figures 4, S4). Cane toads also displayed a broad niche breadth in several environmental variables, which overlapped with most Australian clades, and was higher than observed in most Australian clades (Figure 4; Table S7).

## 4 DISCUSSION

We evaluated niche overlap between the highly invasive cane toad and all native Australian frog species. We examined two important dimensions of the multidimensional niche space: morphology, and the climatic correlates of the environment, in order to discriminate between the empty niche and competitive exclusion hypotheses. Our results demonstrate that while cane toads vary significantly in body size and shape compared to all other Australian frog species, they also occupy a wide ecological niche that overlaps with most frog clades. Therefore, these results suggest that the invasive success of cane toads in Australia may be explained through them successfully colonizing an empty niche among Australian anurans.

Our morphological data strongly support that the invasive cane toads have a unique shape compared to all Australian frog species. Cane toads showed little overlap with any Australian frog species in gross adult body size (PC 1raw) or body shape (PC 2raw and PC 3raw), even considering the effects of phylogenetic correlates. Large body size previously has been suggested to play an important role in amphibian introductions (Tingley et al., 2010), a finding corroborated by our data. This variable was the main predictor for morphological differences with native species, suggesting body size is an important factor contributing to the invasive success of the cane toad. The American bullfrog, *Lithobates catesbeianus,* is another example of an extremely successful amphibian invader that reaches a large adult body size (Snow & Witmer, 2010). Contrastingly, other successful invasive anuran amphibians can reach smaller body sizes, such as the medium‐sized African clawed frog (*Xenopus laevis*), the small‐sized Puerto Rican Coqui frogs (*Eleutherodactylus coqui*), and Greenhouse frogs (*Eleutherodactylus planirostris*). Nevertheless, despite their smaller body size, these invaders most likely also have occupied an empty morphological niche: the African clawed frog is a strictly aquatic pipid species that is substantially morphologically different to native frog species in Europe, and some *Eleutherodactylus* species are highly invasive in Hawaii where no amphibian species occur natively. Furthermore, large body size would likely be an advantageous trait in frogs and toads as it would enable a more generalized diet, higher fecundity, higher mobility, and greater resistance to water loss than smaller species with similar body sizes (Tingley et al., 2010).

Cane toads’ morphology was distinct even after accounting for phylogenetic effects, providing further support for the empty niche hypothesis in an evolutionary context. There was very little morphological niche overlap between cane toads and all the native frog clades in relative limb length ratio (RLLR). This is a good measure of morphological niche position in anuran amphibians, as relative limb proportions are generally highly correlated with the use of structural habitat and locomotive correlates (Enriquez‐Urzelai, Montori, Llorente, & Kaliontzopoulou, 2015; Vidal‐García & Keogh, 2015). Many hylid species occur throughout the cane toads’ current (and potential) distribution (Anstis, 2013; Kearney et al., 2008), and numerous co‐occurring frog species are also ground‐dwelling. Nevertheless, only two genera from the myobatrachid frog radiation had similar RLLR to the cane toad: The sunset frog (*Spicospina* sp.) and toadlets (*Uperoleia* spp.). The sunset frog does not overlap with the cane toad in distribution or habitat (Edwards & Roberts, 2011). In contrast, the species‐rich clade of toadlets (*Uperoleia* spp.) comprises many species that overlap in distribution and ecotype with the cane toad (Anstis, 2013). However, they occupy different microhabitats, and their ecological niche positions are significantly different, probably due to the extreme size difference between adult individual toadlets and cane toads (Anstis, 2013). On the other hand, the Australian hylid frogs of the genus *Cyclorana* have been compared to cane toads due to their ground‐dwelling use of similar structural habitat, their large body sizes, and their diet overlap (Greenlees, Brown, Webb, Phillips, & Shine, 2007), but their contrasting limb morphology may partly explain the lack of competition between these species (Greenlees et al., 2007).

The myobatrachid *Neobatrachus* clade displays relatively similar RLLR and the distributions of most *Neobatrachus* species overlap to some degree with the cane toad, however, their behavior and habitat use differs greatly. *Neobatrachus* spp. are backward burrowers that inhabit a wide range of arid regions, spending most of the time buried and emerging just after heavy rains and flooding (Anstis, 2013). There are no known declines for any species of *Neobatrachus* whose distribution overlaps with that of the cane toad, suggesting the cane toad invasion is having little effect on their realized niche. Differences in niche dimensions between cane toads and morphologically similar native species could lead to different abiotic and biotic interactions, explaining the lack of competition between co‐occurring species from the same ground‐dwelling ecotype. Our finding of strong morphological differentiation between invaders and native species agrees with invasion success, supporting the hypothesis of the empty niche over competitive exclusion, and suggests that successful invasive species display traits that are different from native species (Azzurro et al., 2014; Daehler, 2003; MacDougall et al., 2009).

Besides strong morphological differences with native species, we also found variability within cane toads, especially in size of both males and females. Cane toad populations from their native range also reflect this morphological variability, potentially reflecting differential local adaptation (Hudson, McCurry, Lundgren, McHenry, & Shine, 2016). Recent meta‐analyses in several species of plants, invertebrates, and mammals show that invasiveness success is correlated with trait variability, especially in functionally important morphological traits (Forsman, Wennersten, Karlsson, & Caesar, 2012; González‐Suárez, Bacher, & Jeschke, 2015). Phenotypic plasticity of an invader, coupled with variation of selected traits over time, could lead to niche shifts in one or more dimensions of niche space. This is noticeable in cane toads from the Australian invasion front line, as these individuals have longer hindlimbs than in other populations (Phillips et al., 2006), enabling them to travel much faster and further than other amphibians in the World (Brown, Phillips, & Shine, 2014; Phillips, Brown, & Shine, 2010).

While certain morphological traits can be used to infer the ecological range and habitat use for a given phenotype (Ricklefs & Miles, 1994), some body shapes might work well in multiple environments. The morphological niche position of cane toads differed from native clades, but their environmental and climatic niche breadth overlapped with most Australian frogs, due to their widespread distribution across Australia (Kearney et al., 2008). Thus, cane toads occupy a unique portion of the multidimensional niche space in Australia. A broader ecological niche breadth could reflect higher tolerance of climatic and environmental variation through physiological adaptations that are beneficial in Australia's arid biomes and increasing aridity levels in several areas (Jessop, Letnic, Webb, & Dempster, 2013), thus allowing them to dramatically expand their invasive range in Australia. As such, being able to thrive in a wide range of hostile environments could lead to ecological release, enhancing their invasiveness success (Cadotte, Mcmahon, & Fukami, 2006). Given invasive species could potentially make use of disturbed environments as well as new niches created by anthropogenic changes (Shea & Chesson, 2002), Australian native frog species may be more vulnerable in areas where their preferred microhabitat is not available (Richter‐Boix et al., 2012; San Sebastián, Pujol‐Buxó, Garriga, Richter‐Boix, & Llorente, 2015).

Other factors not related to morphological and environmental niche also might affect the invasiveness potential of the cane toad. For example, successful invaders often are omnivorous, display rapid growth and dispersal, or breed in ephemeral habitats (Cadotte et al., 2006; Ricciardi & Rasmussen, 2011). Cane toads possess all these characteristics, and exhibit a generalist strategy in their trophic niche, which is reflected in the wide environmental and climatic niche breadth. A wide trophic niche breadth, partially due to their large adult body size, would allow cane toads to exploit a wide range of resources, competing with more specialist frogs and potentially displacing them toward different trophic niches (Richter‐Boix et al., 2012; San Sebastián et al., 2015). However, ecological interactions between cane toads and native frog species might be even more complex, due to multiple stages in their life cycle. Physiological, ecological, and behavioral similarities between invasive and native frog tadpoles, as well as a potential breeding overlap with some Australian species, would increase their interactions in both egg and tadpole stages (Crossland et al. 2009; Crossland and Shine 2010).

In addition, differences in morphological traits between juveniles and adults of the invasive cane toad due to ontogenetic allometry could lead to similar morphological niches in juvenile cane toad and adult or juvenile specimens of some Australian species. Thus, this life cycle complexity could result in a niche overlap between cane toad and Australian frogs across different life cycle stages, negatively impacting native species. Furthermore, a lack of natural predators in the invaded areas (Letnic et al., 2008; Shine, 2010), coupled with a fitness advantage (MacDougall et al., 2009) and their lethal toxicity (Letnic et al., 2008), could dramatically favor invasiveness of the cane toad. In addition, the cane toad is the only member of the bufonid family in Australia and is thus very distantly related to native Australian species. Invasive species that are phylogenetically distant from endemic species will be more successful, due to greater niche differentiation and decreased predation (MacDougall et al., 2009; Strauss, Webb, & Salamin, 2006). Thus, due to this taxonomic discordance, Australia might offer lower resistance to alien invasive species than continental regions, by providing the opportunity to invaders to fill an empty niche (Le Breton, Jourdan, Chazeau, Orivel, & Dejean, 2005; Shea & Chesson, 2002; Simberloff, 1995).

Our study is the first to document the significant morphological differences between the invasive cane toad and a continent‐wide frog radiation, supporting the hypothesis that they occupy an empty morphological niche not filled by the native Australian amphibian community. We also propose RLLR (Relative limb length ratio) as a good morphological functional trait in anurans, as it captures information on usage of the structural habitat and locomotive correlates (Vidal‐García & Keogh, 2015; Vidal‐García, Byrne, Roberts, & Keogh, 2014). Cane toad environmental niche breadth is wide, leading to an overlap with most Australian frog clades. Coupled with morphological variation observed within cane toads, as well as behavioral adaptations, this may contribute to invasiveness success. Future research could compare the morphological niche of native species and cane toads in different temporal and spatial populations across Australia, in order to determine whether the morphological niche of cane toads is shifting toward an overlap with native species, which could dramatically impact Australian frog species.

## AUTHOR'S CONTRIBUTIONS

MVG and SK conceived the study. MVG collected and analyzed the data, and drafted the initial version of the manuscript. Both contributed critically to the drafts and gave final approval for publication.

## CONFLICT OF INTEREST

None declared.

## DATA ACCESSIBILITY

Morphological data is available on Dryad (http://dx.doi.org/10.5061/dryad.940m2).
