# Supplementary material for: Invasive cane toads are unique in shape but overlap in ecological niche compared to Australian native frogs
Source: Ecol Evol. 2017 Aug 17;7(19):7609–19. doi: 10.1002/ece3.3253 (PMC5632638; doi:10.1002/ece3.3253)
Supplement: Supplementary file 1 [file ECE3-7-7609-s001.pdf]

Appendix S1

AM: *Adelotus brevis* (77036, 77040, 77042, 80066, 80067), *Assa darlingtoni* (68501, 68502, 68505, 68512, 75004), *Austrochaperina adelphe* (38474, 88681, 88683, 88686, 97898), *A. fryi* (26850, 55079, 58273, 87923, 87925), *A. gracilipes* (62502, 91328, 91336, 91657, 105397), *A. robusta* (87908, 142664, 142665, 145884, 145891), *Crinia bilingua* (72087, 72412, 72422, 72528, 72618), *C. deserticola* (166778, 166883, 166885, 166888, 166896), *C. georgiana* (145908, 148905), *C. glauerti* (6757, 7701), *C. insignifera* (104375, 104376, 104378, 104387, 123431), *C. parasignifera* (29469, 34226, 34332, 34778, 71872), *C. riparia* (37710, 57174, 96480, 96485, 96488), *C. signifera* (110770, 110897, 112412, 112948, 112949), *C. sloanei* (141836), *C. tasmaniensis* (10342, 10343, 12329, 10349.1, 10349.2), *C. tinnula* (160106, 165603, 171074, 171080, 171093), *Cyclorana australis* (10147, 30122, 32058, 60334, 97362), *C. brevipes* (11700, 28193, 53899, 150852, 152939), *C. longipes* (97379), *C. maculosa* (60328), *C. maini* (121149, 140508), *C. novaehollandiae* (38187, 38188, 38191, 80739, 82554), *C. platycephala* (152821, 152823, 152882, 152971, 152972), *C. verrucosa* (173114, 173116), *Geocrinia laevis* (1591, 1598, 1599, 1600, 102936), *G. leai* (39293, 39295, 39297, 39334, 39336), *G. victoriana* (75872, 98151, 108145, 108563, 108567), *Heleioporus albopunctatus* (38344, 39180, 39181, 39376), *H. australiacus* (27631, 29447, 78853, 99416, 99484), *H. eyrei* (19215, 19216, 38372, 69174, 69187), *H. psammophilus* (39216, 39218, 39220), *Lechriodus fletcheri* (141657, 141662, 141663, 141664, 143432), *Limnodynastes convexiusculus* (82255), *L. dorsalis* (2993, 7453, 7455, 7586), *L. dumerilii* (162946, 168497, 171119, 171924), *L. fletcheri* (121048, 142800, 164826, 164827, 173109), *L. interioris* (125427, 153879, 156877, 174819, 174820), *L. peronii* (153422, 153899, 156300, 158418, 158460), *L. salmini* (36158, 73567, 78890, 138793, 155912), *L. tasmaniensis* (167569, 167571, 171178, 174205, 174236), *L. terraereginae* (133220, 133221, 139409, 146696, 146731), *Litoria adelaidensis* (160626, 160637, 160648), *L. aurea* (71699, 74546, 74550, 89809, 89810167115), *L. barringtonensis* (144718, 147504, 74026, 139343, 144717), *L. bicolor* (46529, 46541, 46822, 46921, 48281), *L. booroolongensis* (36304, 36496, 36521, 36525, 36544), *L. brevipalmata* (37456), *L. burrowsae* (15886), *L. caerulea* (18477, 73983, 73985), *L. castanea* (32183, 34937, 36430, 51295, 51700), *L. cavernicola* (12362, 126196), *L. chloris* (37423, 37429, 41364, 71244, 103073), *L. citropa* (76625, 79100, 79437, 130673, 155625), *L. dahlii* (27098, 38676, 38918, 41333), *L. eucnemis* (114622, 114637, 114654, 114681, 114807), *L. ewingii* (117994, 165304, 165322, 165323, 175372), *L. fallax* (132669, 144880, 148861, 164168, 175374), *L. freycineti* (78678, 78679, 78681, 78911, 78913), *L. gilleni* (73632, 73641, 73642, 73646, 111193), *L. infrafrenata* (31.393, 31394, 56237, 82248, 132195), *L. jerviensis* (38278, 54005, 78793, 78796, 84882), *L. jungguy* (87986, 87987, 87988, 87989), *L. latopalmata* (31822, 32198, 32429, 32434, 33918), *L. lesueurii* (10299, 12657, 12658, 12659, 12662), *L. littlejohni* (25999, 97775, 97777, 145523, 161383), *L. moorei* (39337, 69191, 69192, 102265), *L. nannotis* (11529, 53892, 56420, 58272), *L. nigrofrenata* (91367, 91463, 91465, 99942, 121160), *L. nudigitus* (70004, 108664, 108779, 108781, 108786, 108786), *L. peronii* (103654, 105945, 119746, 119748, 119863), *L. personata* (39804, 39805, 75644), *L. phyllochroa* (78949, 79386, 79391, 79396, 139506), *L. raniformis* (102857, 102858, 102860, 102930, 102935), *L. spenceri* (145932), *L. splendida* (123820, 123845), *L. subglandulosa*

(42933, 51097, 51736, 51736, 51737, 52630), *L. verreauxi* (69746, 71477, 71668, 71668, 77178, 80309, 166537, 166549, 166551, 166584, 166594), *L. xanthomera* (53885, 143300), *Metacrinia nichollsi* (98288), *Mixophyes hihihorlo* (120835, 120836, 120838, 120840, 120841), *Neobatrachus sudelli* (152817, 152846, 152929, 152978, 152979), *Notaden bennetti* (45617, 45618, 45623, 45628, 156793), *N. melanoscaphus* (53575, 53583, 53584, 53703), *N. nichollsi* (49446, 49447, 49455, 49458, 110616), *Nyctimystes dayi* (26603, 26779, 39722, 53954, 61388), *Paracrinia haswelli* (55022, 162984, 171039, 171040), *Philoria frosti* (78660), *P. kundagungan* (145667, 165002, 165134, 165135), *P. loveridgei* (68526, 75013, 86326, 131927, 165039), *P. pughi* (96794, 132319, 137708, 137708), *P. richmondensis* (130992, 132432, 132454, 132457), *P. sphagnicolus* (165051), *Pseudophryne australis* (150131, 153491, 154030, 161743, 165552), *P. bibronii* (158017, 164934, 165325, 167678, 171042), *P. coriacea* (145705, 146199, 151296, 163068, 163071), *P. corroboree* (76234, 144854, 144855), *P. covacevichae* (17037), *P. dendyi* (27727, 29860, 71425, 78970), *P. guentheri* (1533, 11147, 19503, 19504, 71261), *P. pengilleyi* (71261), *P. raveni* (53794), *P. semimarmorata* (9829, 14445, 28207, 75853, 75860), *Rana daemeli* (122007, 122007, 122008, 122009, 122012, 122014), *Rhinella marina* (107577, 107856, 107856, R105051, R105097, R105156, R107090, R107576, R107577, R107578, R107583, R107585, R107586, R107828, R107830, R107831, R107832, R107843, R107844, R107845, R107854, R107855, R107856, R107857, R12257, R12259, R16373, R16374, R16384, R17110, R174792, R174877, R517111), *Taudactylus acutirostris* (53865, 53866, 53868, 94449), *T. diurnus* (41218, 78716, 127407, 127408), *T. eugellaensis* (77190, 77208, 77210, 89076, 113900), *T. liemi* (47527), *T. rheophilus* (56477, 58271); **NT**: *Rhinella marina* (246, 247, 5511, 19184, 20523, 20535, 21321, 21716, 26280, 26281, 27193, 27194, 27195, 28036, 29267, 29708, 29710, 34931, 35389, 88719, 91398); **QM**: *Austrochaperina pluvialis* (28816, 32148, 32149, 53988, 55537), *Cophixalus aenigma* (25302, 53859, 53862, 53905, 55489), *C. australis* (39923, 43655, 61251, 65377, 70701), *C. bombiens* (56524, 56529), *C. concinnus* (42263, 43899, 43917, 78449, 79448), *C. crepitans* (32451, 32471, 41647, 70604, 70605), *C. hosmeri* (55903, 79535, 27055, 55907, 55916), *C. infacetus* (48792, 55557, 55558, 65712, 68224), *C. kulakula* (88503, 88539, 88541, 88542, 88543), *C. macdonaldi* (29271, 47213, 51662, 52817, 59694), *C. monticola* (58729, 58730, 58854, 58857, 58873), *C. neglectus* (39919, 41868, 55798, 56500, 62957), *C. ornatus* (61976, 61978, 66967, 66968, 71286), *C. pakayakulangun* (88545, 88546, 88547, 88548), *C. peninsularis* (42061, 42062), *C. saxatilis* (28773, 48798, 48799, 57834, 64888), *Cyclorana alboguttata* (90817), *C. manya* (57703, 65746, 66703, 66705, 91234), *C. verrucosa* (56817, 85888, 85889), *Litoria andiirrmalin* (59000, 59001, 59002, 59003, 83178), *L. burrowsae* (37959), *L. cooloolensis* (31347, 35580, 59586, 61965, 64787), *L. electrica* (81901, 81902, 90107, 90108, 90110), *L. longirostris* (84430), *L. lorica* (75756, 86823), *L. myola* (82420, 82421, 82422, 82426, 82427), *L. nyakalensis* (55592, 55595, 55770, 55776, 55786), *L. olongburensis* (34273, 59607, 62044), *L. paraewingi* (29809, 29839, 29840), *L. revelata* (56571, 56572, 56574, 56575, 72762), *L. rheocola* (41286, 54449, 54451, 56579, 60951), *L. xanthomera* (54965, 54976, 66043), *Mixophyes coggeri* (53691), *Philoria richmondensis* (27772), *Pseudophryne covacevichae* (53920, 53925, 53929, 53931), *P. raveni* (42644, 42645, 42648, 42669), *Taudactylus liemi* (31515, 31516, 32622), *T. pleione* (42137, 42388, 42389, 42391, 42422); **SAM**: *Cophixalus bombiens* (35810, 35811, 35812), *C. exigus* (9796, 9832, 10035,

10036, 10037), *C. saxatilis* (34324), *C. zweifeli* (51080), *Crinia georgiana* (3956, 16509A, 5994B), *C. glauerti* (5971, 5992, 5967C), *C. nimbus* (C1078, C1080A, C1080B, C1085), *C. pseudinsignifera* (16508A, 16508D, 16508E), *C. remota* (42242, 42243, 42245, 44155, 57482), *C. sloanei* (22414, 30930, 30939, 30942), *C. subinsignifera* (30428), *Cyclorana alboguttata* (63680, 63687, 63688, 63689), *C. maculosa* (20344, 43430, 43432, 63718, 63720), *C. maini* (46964, 46974, 46975, 47081, 49326), *Heleioporus albopunctatus* (10183), *H. barycragus* (5975, 42318, 26932, 26933, 26935), *H. inornatus* (26937, 26938, 26940, 26946), *H. psammophilus* (5968, 42303), *Limnodynastes convexiusculus* (41981, 41985, 14234A, 14234B), *L. depressus* (23862, 23863, 23864, 16889A, 16889C), *L. dorsalis* (32413), *L. dumerilii* (71754), *Litoria adelaidensis* (63241, 63243), *L. brevipalmata* (133423, 13342A, 13342C, 13342D), *L. burrowsae* (9730A), *L. cyclorhyncha* (32409, 63235, 63287), *L. daviesae* (51054, 51055, 51056, 51059, 51060), *L. dentata* (12204, 40858, 13305A, 13305C, 13341A), *L. genimaculata* (20238, 34583, 34656, 38632, 65025), *L. gracilenta* (34600, 34911, 63692), *L. longirostris* (15221, 15222, 42604, 42605), *L. lorica* (17349, 17350, 17351), *L. microbelos* (25459, 43616A, 43616B, 43616C, 43616D), *L. olongburensis* (40212, 40213), *L. paraewingi* (44066, 44066, 44074, 44077), *L. personiana* (12625, 12628, 17584, 17585, 43693), *L. piperata* (13885, 13886, 13887, 13889), *L. tyleri* (12250, 17555, 32576, 32577, 32579), *L. wilcoxii* (66179, 66256, 66257, 66258, 66259), *Mixophyes balbus* (12294, 12295, 12297, 19812, 59921), *M. carbinensis* (64487, 64489, 64491, 64492, 64495), *M. coggeri* (64482, 64483, 64484, 64485), *M. fasciolatus* (40310, 40311, 40312, 51181, 51182), *M. fleayi* (59930, 59931, 59934, 65429, 65430), *M. iteratus* (12287, 12289, 12290, 12291, 51193), *M. schevilli* (19804, 38613, 38615, 38616, 43753), *Neobatrachus aquilonius* (5086A, 5086B, 5086D, 5087A, 5087C), *N. fulvus* (50673), *N. inornatus* (5990), *N. kunapalari* (5965, 7534, 7535, 37865, 50560), *N. pelobatoides* (5980, 17769, 37866, 37878, 5984B), *N. pictus* (7275, 18055, 18057, 18060, 18061), *N. sutor* (23994, 23996, 23997, 32399, 40590), *N. wilsmorei* (37870, 5978A, 5978B), *Notaden melanoscaphus* (17904), *Paracrinia haswelli* (71996), *Philoria frosti* (9598, 19045, 19371, 19376), *P. kundagungan* (39204), *P. pughi* (57827), *P. sphagnicolus* (10407, 57823, 57824, 57825), *Pseudophryne corroboree* (13474, 4262), *P. dendyi* (13758), *P. guentheri* (13876), *P. major* (39896, 39898, 39899, 39915, 39920), *P. occidentalis* (14399.1, 14399.2, 14399.3, 14399.4, 14399.4), *P. pengilleyi* (44410, 44412, 44416, 44461), *P. robinsoni* (11740, 11742, 58751, 64657, 13276D), *Rheobatrachus silus* (34077, 34081, 34086, 34087, 34101), *R. vitellinus* (49712, 49715, 49716, 49717, 58747), *Spicospina flammocaerulea* (46671), *Taudactylus acutirostris* (20256), *T. diurnus* (13963A), *T. liemi* (34661), *T. rheophilus* (34596, 34598, 39258), *Uperoleia altissima* (49739, 49740, 49741, 49742, 49743), *U. arenicola* (16691, 16992, 16993, 16994, 16996), *U. aspera* (28788, 28789, 28791, 38001, 49754), *U. borealis* (23834, 29698, 29700, 29701, 29702), *U. capitulata* (29549, 29592, 29593, 29595, 34443), *U. crassa* (28854, 28858, 48886, 48887, 48901), *U. daviesae* (65273, 65275, 65280, 65286, 65287), *U. fusca* (33926, 33927, 39814, 43769, 43770), *U. glandulosa* (27081), *U. inundata* (25772, 28663, 28699, 28731, 43731), *U. lithomoda* (24343, 28766, 28767, 28769, 29695), *U. littlejohni* (39801, 39805, 39807, 39808, 39809), *U. martini* (64590, 67393, 67394, 67395, 67396), *U. micromeles* (17175, 17176, 17177, 17178), *U. mimula* (30370, 30378, 30416, 34320, 34322), *U. minima* (17085, 17086, 17087, 17088, 17089), *U. mjoebergii* (18021, 18022, 18023, 18029, 28869), *U. rugosa* (18882, 25012, 25082, 55117, 71690), *U. russelli*

(17232, 34699, 34700, 34704, 34706), *U. talpa* (28807, 28817, 28819, 28831, 31733), *U. trachyderma* (22334, 22339, 22341, 25956, 25958); **WAM***:* *Arenophryne rotunda* (165795, 165798, 165801, 165806, 165808), *A. xiphorhynca* (123493, 123523, 123556, 123566, 126244), *Crinia frimbiata* (163823, 167744, 167745), *C. subinsignifera* (131289, 138700, 138703, 138704), *Cyclorana cryptotis* (71010, 71012, 162458, 162462, 164728), *C. cultripes* (73461, 87173, 87174, 87181, 89984), *C. longipes* (164622, 164623, 164678, 164767), *C. maini* (162418, 163034, 163057), *C. vagita* (164693, 164694, 164729, 164945, 164946), *Geocrinia alba* (86527, 95958, 95959, 95983, 163641), *G. lutea* (86528, 86537, 101093, 144385), *G. rosea* (123397, 123449, 152138, 164151, 164153), *G. vitellina* (86475, 86476, 86477, 86482, 95984), *Limnodynastes lignarius* (171449, 171479, 171480, 171511, 171963), *Litoria aurifera* (16800, 168003, 168058, 168063, 168128), *L. axillaris* (171471, 171473), *L. caerulea* (164645, 164646, 164855), *L. cavernicola* (164903, 167793, 168188), *L. coplandi* (162520, 167757, 167784, 167785, 167993), *L. cyclorhyncha* (17572, 17611), *L. dahli* (34601), *L. gracilenta* (68282, 146065), *L. inermis* (164708, 164709, 166014, 167870, 167871), *L. meiriana* (156238, 162525, 162527, 162600, 164790), *L. microbelos* (43366, 43370), *L. moorei* (144372), *L. nannotis* (42439), *L. nasuta* (164887, 164954, 167705, 167707, 167834), *L. pallida* (73577, 87031, 87300, 87301, 94325), *L. rothii* (136156, 140386, 140790, 140792, 140794), *L. splendida* (168082, 168083, 171962), *L. staccato* (167737, 167738, 167936, 168068, 171469), *L. tornieri* (53784, 53785, 53786, 167779, 167828), *L. wotjulumensis* (168054, 168075, 168122, 168165, 171464), *Metacrinia nichollsi* (57406, 90141, 95258, 95265), *Myobatrachus gouldii* (146470, 146470, 146471, 149224, 149541, 150222), *Neobatrachus albipes* (52547, 65135, 91158, 94282, 136419), *N. fulvus* (52938, 52942, 76583, 87361), *N. wilsmorei* (145152, 145155, 145157), *Notaden weigeli* (164374, 164901, 164915, 164916, 164942), *Platyplectrum ornatum* (129117, 129120, 129121, 129122, 129225), *P. spenceri* (123856, 123863, 154581, 154583, 154590), *Pseudophryne douglassi* (69595, 69608, 102392, 125741, 138094), *Spicospina flammocaerulea* (112150, 119458, 119459, 144371), *Uperoleia glandulosa* (99924, 100597, 135895, 154273), *U. laevigata* (RAC0005, RAC0006, RAC003 & two new specimens (without a code) - to be added to the WAM collection), *U. micromeles* (127139), *U. saxatilis* (22908, 22916, 39231, 63110, 68945), *U. tyleri* (RAC001, RAC0080, RAC0081, RAC0083, RAC0084 - to be added to the WAM collection), *U. variegata* (62462, 62463, 62464, 62470, 62471)
