# Supplementary material for: Invasive cane toads are unique in shape but overlap in ecological niche compared to Australian native frogs
Source: Ecol Evol. 2017 Aug 17;7(19):7609–19. doi: 10.1002/ece3.3253 (PMC5632638; doi:10.1002/ece3.3253)
Supplement: Supplementary file 2 [file ECE3-7-7609-s002.docx]

**Appendix S2**

**Materials and methods**

We used principal components analysis (PCA) to reduce the dimensionality of the morphological data set for 24 variables corrected by body size (residuals of the linear regression between all raw morphological variables and SVL). We performed an analysis of variance (ANOVA) test on the first three PC for the size-corrected morphological PCA (PC 1sc, PC 2sc, PC 3sc) among each of the Australian frog genera and clades. We performed a phylogenetic ANOVA for both univariate and multivariate data in *geomorph* (Adams 2014b), to test whether phylogeny affected size-corrected morphological traits (size-corrected as per Revell, 2009). We also performed phylogenetic regression models using this function to test the correlation between sets of traits. We then performed a size-corrected phylogenetic PCA on all morphological variables, with *phytools* (Revell 2012), as per Revell (2009).

**Results**

The first principal component for the size-corrected morphological data set (PC 1sc) accounted for 35.37 % of the variance, and PC 2sc and PC 3sc accounted for 17.64 % and 9.1 %, respectively. PC 1sc was correlated with relative tibial length (Tibial l./SVL: R^2^_adj_ = 0.8624, p < 0.0001; Fig. S2; Table S3) and relative femur length (Femur l./SVL: R^2^_adj_ = 0.8172, p < 0.0001), PC 2sc was mostly correlated with relative mouth width (Mouth w./SVL: R^2^_adj_ = 0.6476, p < 0.0001) and relative head width (Head w./SVL: R^2^_adj_ = 0.6153, p < 0.0001), and PC3 corresponded to pointiness of the snout (Naris-snout l./SVL: R^2^_adj_ = 0.4101, p < 0.0001). For the size-corrected phylogenetic PCA, the first principal component (PCp 1phy_sc) accounted for 59.44 % of the variance, whereas PCp 2phy_sc and PCp 3phy_sc explained 14.72 % and 7.52 % of the morphological variability, respectively (Fig. S3AB; Table S4). PCp 1phy_sc correlated strongly with foot length (phylo-residuals of foot l. on SVL: R^2^_adj_ = 0.9511, p < 0.0001), tibial length (R^2^_adj_ = 0.9478, p < 0.0001), and femur length (R^2^_adj_ = 0.8791, p < 0.0001). PCp 2phy_sc was correlated with head width (R^2^_adj_ = 0.8808, p < 0.0001) and mouth width (R^2^_adj_ = 0.8712, p < 0.0001), and PCp 3phy_sc with feet webbing (R^2^_adj_ = 0.5442, p < 0.0001). ANOVAs and Dunnett’s tests also depicted differences between Australian frogs and cane toads in size-corrected morphology (F_45, 1170_ = 97.16, p < 0.0001 and F_1, 1214_ = 38.28, p < 0.0001 for PC 1sc; F_45, 1170_ = 57.23, p < 0.0001 and F_1, 1214_ = 13.35, p = 0.0003 for PC 2sc; and F_45, 1170_ = 104.9, p < 0.0001 and F_1, 1214_ = 76.91, p < 0.0001 for PC 3sc; Table S5, Fig. S2).
