# Supplementary figures and images for: Invasive cane toads are unique in shape but overlap in ecological niche compared to Australian native frogs
Source: Ecol Evol. 2017 Aug 17;7(19):7609–19. doi: 10.1002/ece3.3253 (PMC5632638; doi:10.1002/ece3.3253)

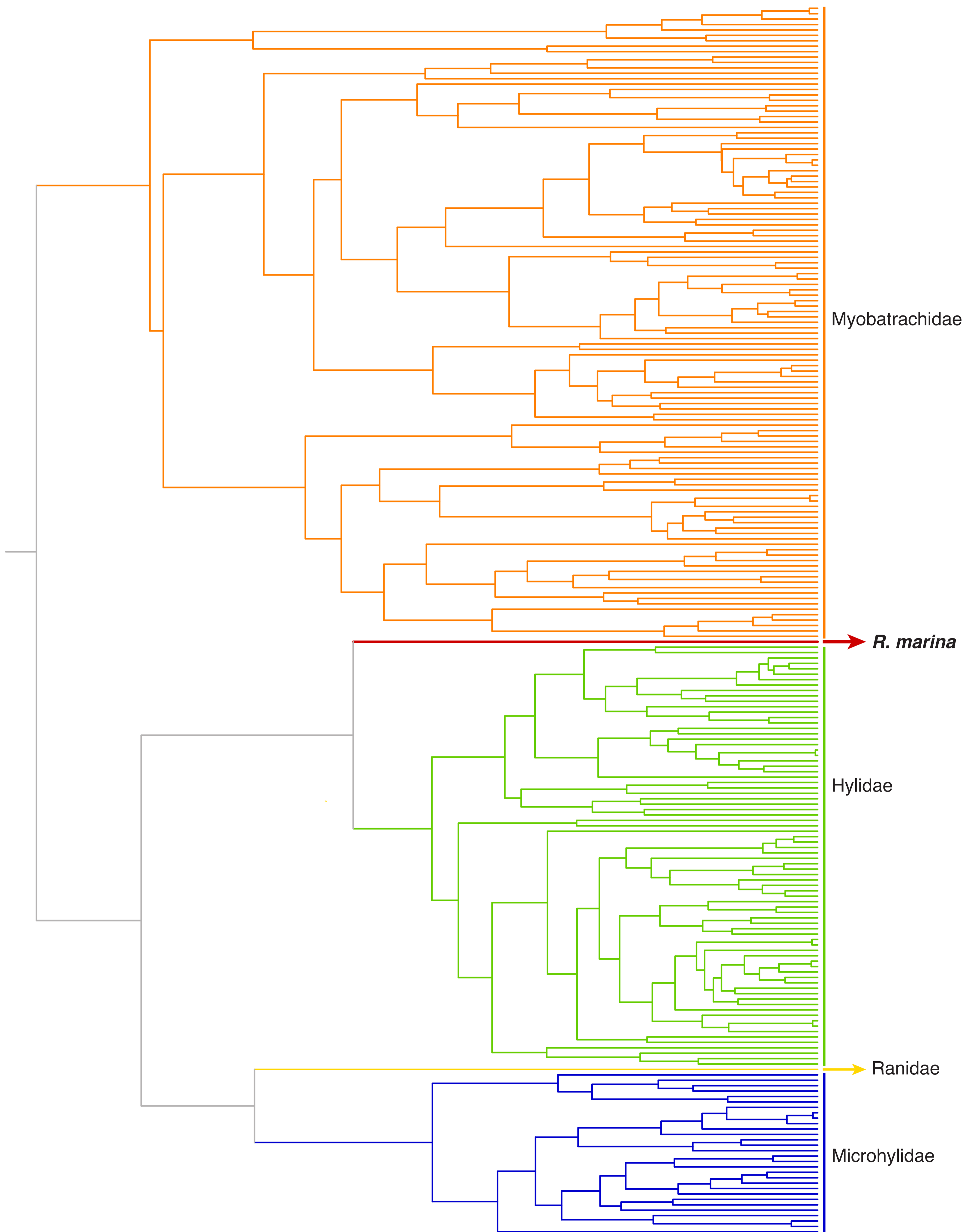

Supplement: Supplementary file 3 [file ECE3-7-7609-s003.pdf]

PC 2sc

10

0

-10

- Microhylidae
- Ranidae
- Hylidae
- Bufo
- Myobatrachidae

-25

0

25

PC 1sc

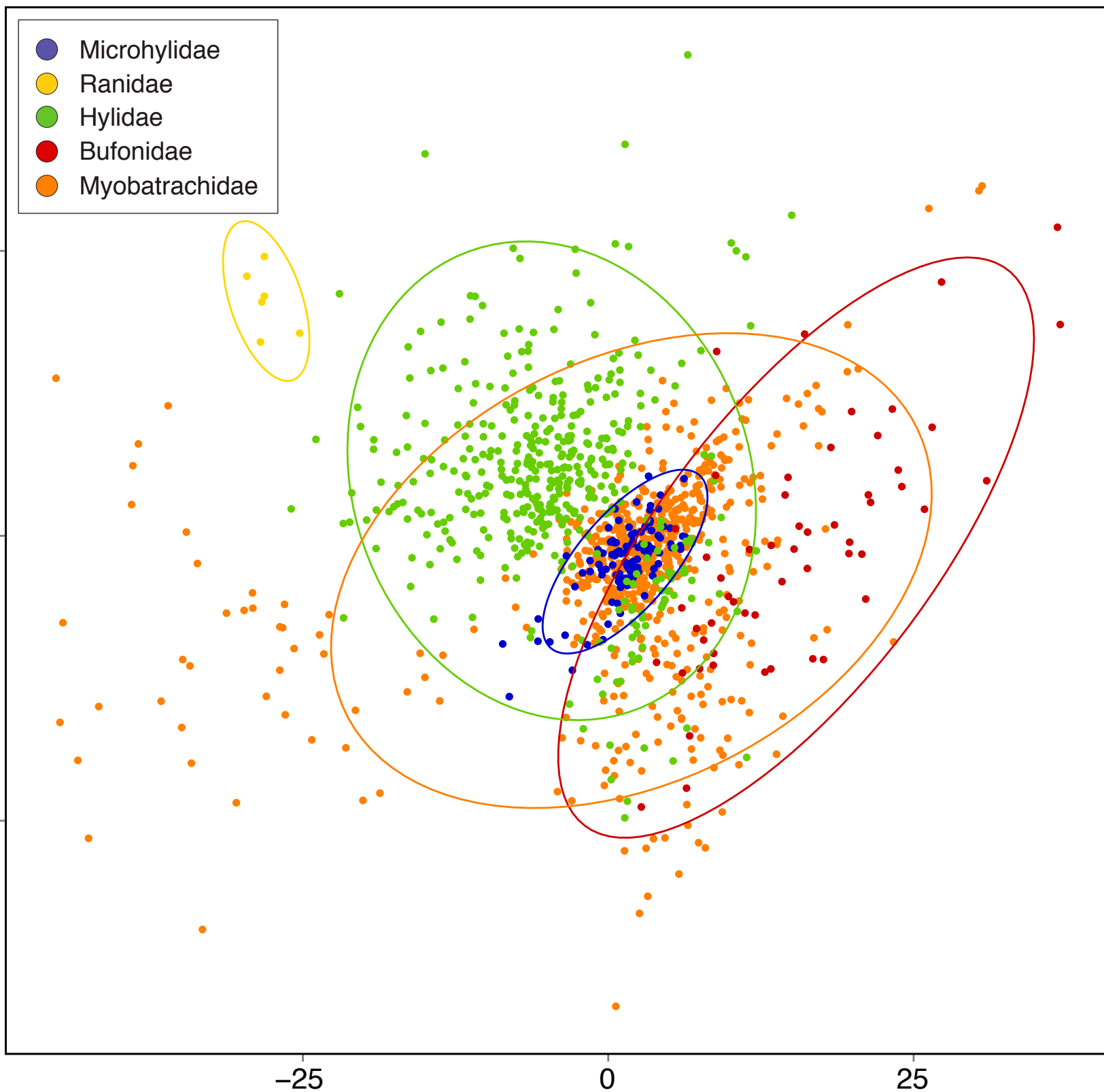

Supplement: Supplementary file 4 [file ECE3-7-7609-s004.pdf]

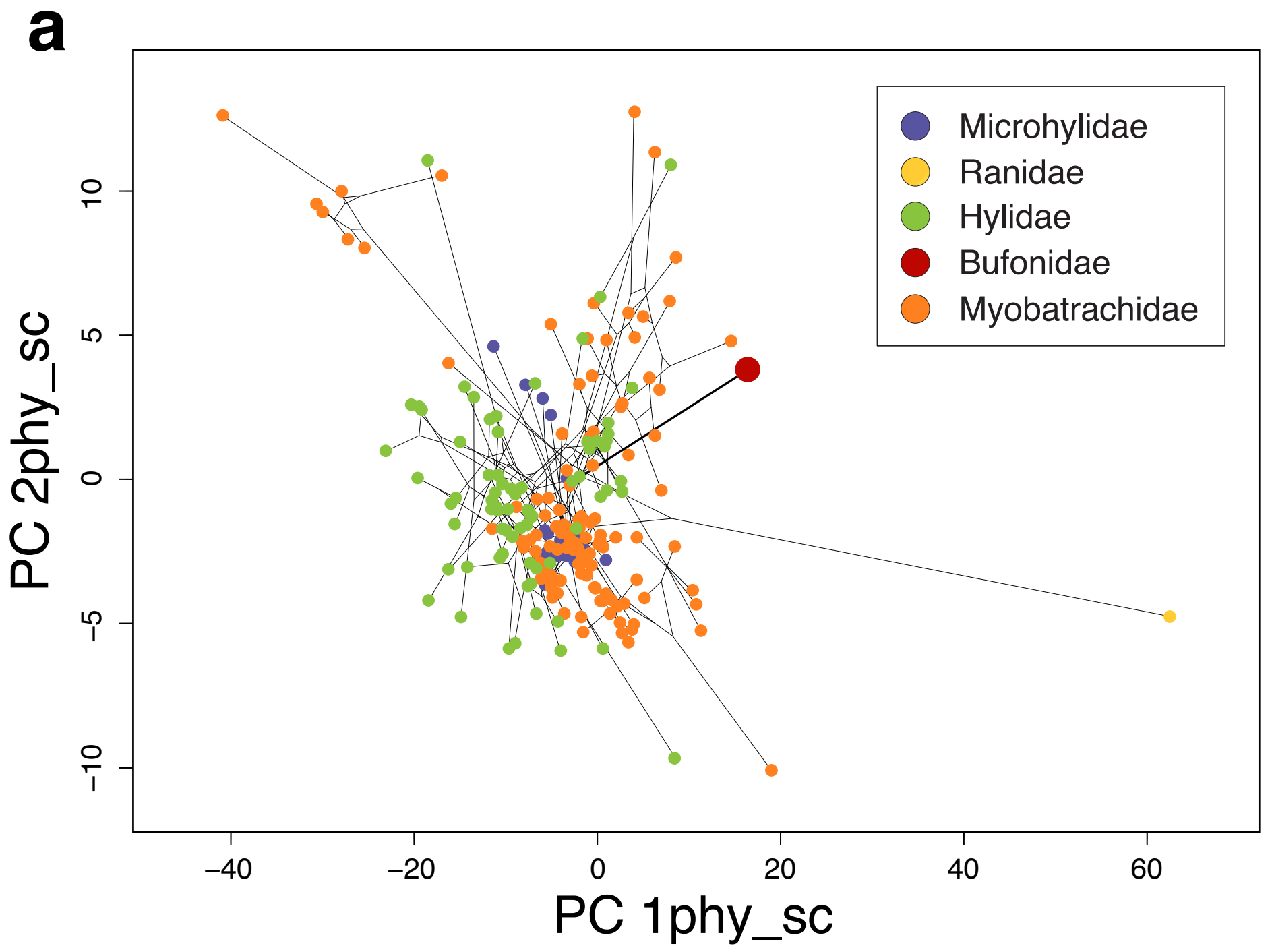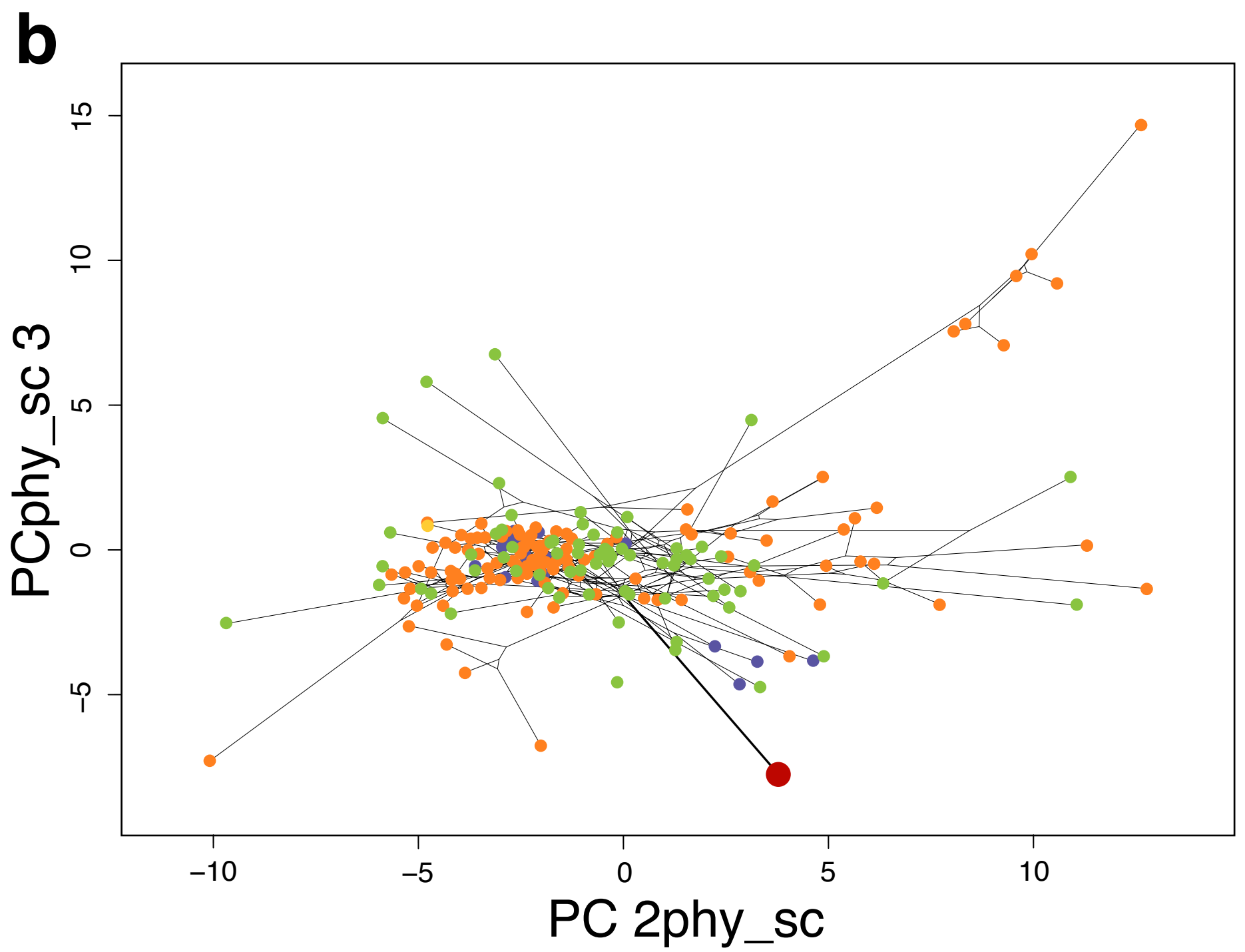

Supplement: Supplementary file 5 [file ECE3-7-7609-s005.pdf]

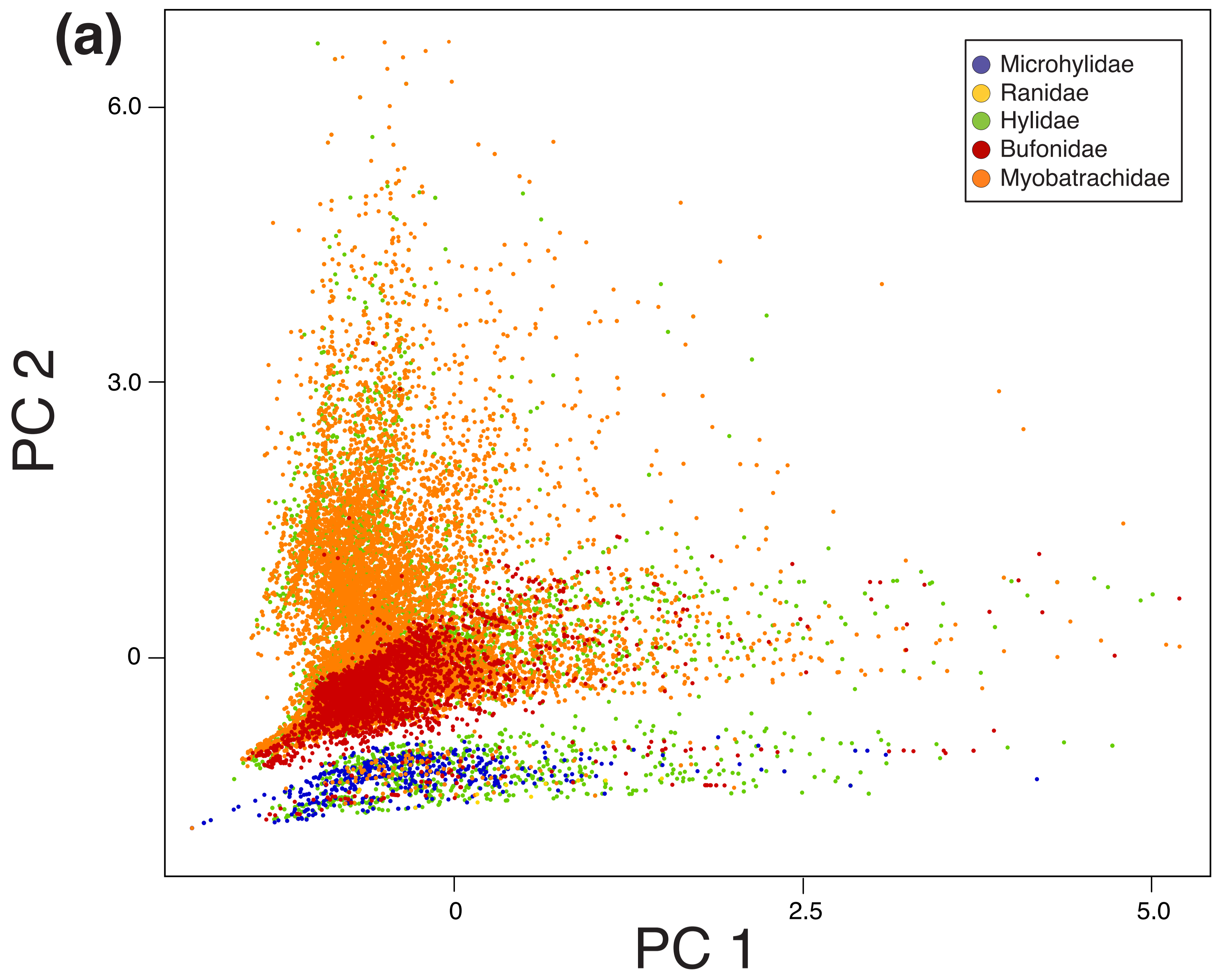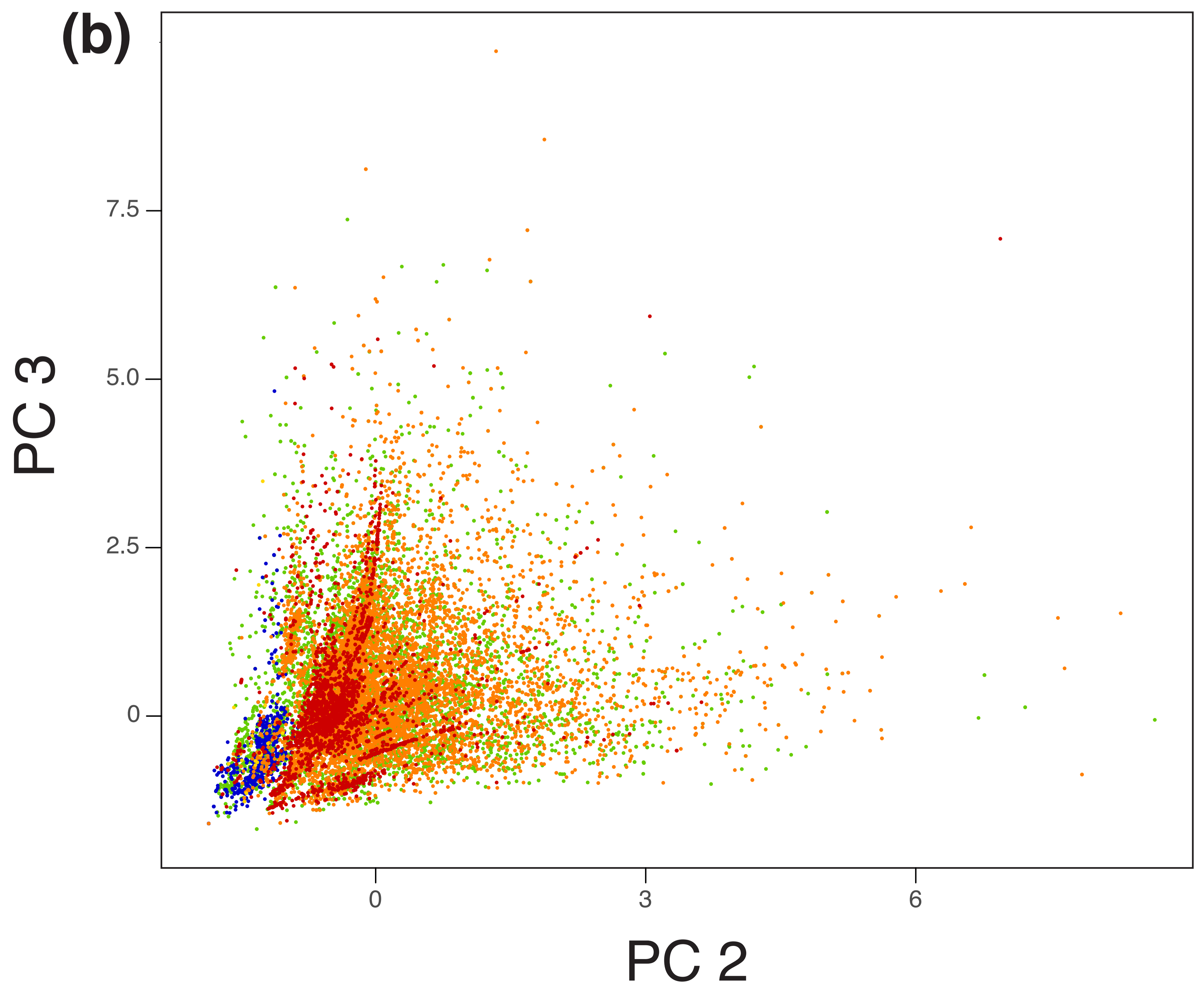

Supplement: Supplementary file 6 [file ECE3-7-7609-s006.pdf]
