# Supplementary material for: Invasive cane toads are unique in shape but overlap in ecological niche compared to Australian native frogs
Source: Ecol Evol. 2017 Aug 17;7(19):7609–19. doi: 10.1002/ece3.3253 (PMC5632638; doi:10.1002/ece3.3253)
Supplement: Supplementary file 7 [file ECE3-7-7609-s007.docx]

| Table S1. PCA loadings for the morphological dataset, using 25 raw variables. | | | | | |
| --- | --- | --- | --- | --- | --- |
|  |  |  |  |  |  |
| **Axis** | **1** | **2** | **3** | **4** | **5** |
| **Eigenvalues (λ)** | **22.40043** | **1.33059** | **0.32739** | **0.19994** | **0.14771** |
| **Total variance explained (%)** | **89.60173** | **5.32238** | **1.30955** | **0.79975** | **0.59084** |
| SVL | 0.97715 | -0.16331 | -0.05941 | 0.04245 | 0.02556 |
| Head length (jaw) | 0.98587 | -0.03617 | 0.06903 | -0.05322 | 0.04656 |
| Head width | 0.97605 | -0.14866 | 0.05848 | -0.04776 | 0.08624 |
| Eye-naris distance | 0.94649 | 0.14710 | -0.07729 | -0.18777 | 0.10190 |
| Interorbital span | 0.97332 | -0.02255 | -0.07665 | -0.13470 | 0.08449 |
| Internarial span | 0.95814 | -0.01921 | 0.15873 | -0.14008 | -0.06248 |
| Naris-Snout distance | 0.90536 | 0.11381 | 0.33617 | -0.14878 | -0.09238 |
| Eye length | 0.93943 | -0.25306 | 0.07325 | 0.00518 | 0.06622 |
| Mouth width | 0.97478 | -0.18312 | 0.05224 | -0.00316 | 0.03680 |
| Humerus length | 0.96044 | -0.23304 | -0.00503 | 0.06093 | 0.00151 |
| Forearm length | 0.96074 | -0.23603 | -0.02452 | 0.04096 | -0.00655 |
| Wrist width | 0.94197 | -0.27585 | -0.02395 | 0.07547 | 0.07141 |
| Hand length | 0.98581 | -0.02708 | -0.13007 | -0.01233 | 0.01711 |
| Thumb length | 0.96998 | -0.00598 | -0.11174 | -0.00409 | -0.02366 |
| Finger 4 length | 0.92106 | 0.25556 | -0.23616 | -0.08140 | -0.01393 |
| Femur length | 0.98837 | 0.08459 | -0.01220 | 0.00142 | -0.01947 |
| Femur width | 0.94018 | -0.21341 | 0.06269 | 0.16329 | 0.03062 |
| Tibial length | 0.96838 | 0.19589 | -0.01058 | -0.00054 | -0.07114 |
| Tibial width | 0.96242 | -0.16140 | 0.04135 | 0.07481 | 0.04943 |
| Foot length (toe 1) | 0.98297 | 0.07254 | 0.04122 | 0.06503 | -0.07555 |
| Foot length (total) | 0.99157 | 0.06492 | -0.02037 | 0.01548 | -0.05854 |
| Toe 1 length | 0.91056 | 0.31391 | -0.19040 | -0.00074 | -0.02733 |
| Toe 5 length | 0.85929 | 0.47904 | 0.09414 | 0.07181 | -0.00334 |
| Webbing 4-5 length | 0.71141 | 0.64477 | 0.10931 | 0.18510 | 0.10866 |
| Elbow-axilla length | 0.93044 | -0.15794 | -0.07788 | 0.06161 | -0.25503 |
