# Supplementary material for: Invasive cane toads are unique in shape but overlap in ecological niche compared to Australian native frogs
Source: Ecol Evol. 2017 Aug 17;7(19):7609–19. doi: 10.1002/ece3.3253 (PMC5632638; doi:10.1002/ece3.3253)
Supplement: Supplementary file 8 [file ECE3-7-7609-s008.docx]

| Table S2. PCA loadings for the morphological dataset, using the residuals of the linear regression of each of the 24 morphological raw variables against SVL | | | | | |
| --- | --- | --- | --- | --- | --- |
|  |  |  |  |  |  |
| **Axis** | **1** | **2** | **3** | **4** | **5** |
| **Eigenvalues (λ)** | **9.77530** | **3.67370** | **2.90744** | **2.23043** | **1.76224** |
| **Total variance explained (%)** | **67.36000** | **9.51300** | **5.95800** | **3.50700** | **2.18900** |
| Head length (jaw) | -0.15784 | -0.34277 | -0.17178 | 0.18044 | -0.37015 |
| Head width | -0.06557 | -0.48261 | -0.23695 | 0.22417 | -0.03439 |
| Eye-naris distance | -0.06010 | -0.01349 | -0.02302 | 0.17236 | -0.02040 |
| Interorbital span | -0.07165 | -0.08024 | -0.04170 | 0.34263 | 0.15361 |
| Internarial span | -0.04496 | -0.08608 | -0.02453 | 0.02718 | -0.03700 |
| Naris-Snout distance | -0.04456 | -0.06610 | -0.02574 | -0.03599 | -0.10793 |
| Eye length | 0.00302 | -0.16862 | -0.04197 | -0.01543 | 0.07851 |
| Mouth width | -0.04660 | -0.52967 | -0.18608 | 0.01913 | 0.01590 |
| Humerus length | 0.02148 | -0.27232 | 0.00470 | -0.23515 | 0.43995 |
| Forearm length | 0.00792 | -0.19610 | 0.01460 | -0.09387 | 0.34844 |
| Wrist width | 0.01921 | -0.06553 | -0.01699 | -0.03444 | 0.08947 |
| Hand length | -0.10949 | 0.01145 | 0.07547 | 0.28710 | 0.38609 |
| Thumb length | -0.06660 | 0.02028 | 0.02955 | 0.09404 | 0.14915 |
| Finger 4 length | -0.12994 | 0.14297 | 0.06601 | 0.31211 | 0.15142 |
| Femur length | -0.36182 | -0.05283 | 0.02398 | 0.17508 | -0.02237 |
| Femur width | 0.00926 | -0.11106 | -0.00799 | -0.36346 | -0.24014 |
| Tibial length | -0.52510 | 0.09101 | 0.17994 | 0.10672 | -0.24074 |
| Tibial width | -0.01451 | -0.08173 | -0.01550 | -0.10322 | -0.08942 |
| Foot length (toe 1) | -0.30196 | -0.10828 | 0.15597 | -0.50094 | 0.07043 |
| Foot length (total) | -0.47461 | -0.07956 | 0.31600 | -0.13968 | 0.25162 |
| Toe 1 length | -0.10949 | 0.11915 | 0.05465 | 0.11952 | 0.03606 |
| Toe 5 length | -0.33698 | 0.10898 | -0.13026 | -0.10748 | -0.22356 |
| Webbing 4-5 length | -0.26958 | 0.32012 | -0.82726 | -0.16059 | 0.23569 |
| Elbow-axilla length | 0.00768 | 0.08736 | 0.03499 | 0.08117 | 0.04395 |
