# Supplementary material for: Invasive cane toads are unique in shape but overlap in ecological niche compared to Australian native frogs
Source: Ecol Evol. 2017 Aug 17;7(19):7609–19. doi: 10.1002/ece3.3253 (PMC5632638; doi:10.1002/ece3.3253)
Supplement: Supplementary file 9 [file ECE3-7-7609-s009.docx]

| Table S3. Phylogenetically-corrected PCA for all raw variables, using *phytools*. | | | | | |
| --- | --- | --- | --- | --- | --- |
|  |  |  |  |  |  |
| **Axis** | **1** | **2** | **3** | **4** | **5** |
| **Eigenvalues (λ)** | **25081.81000** | **533.27840** | **131.68560** | **98.11952** | **56.65286** |
| **Total variance explained (%)** | **96.10015** | **2.04324** | **0.50455** | **0.37594** | **0.21706** |
| SVL | -0.99251 | -0.09776 | 0.06286 | -0.03439 | 0.00066 |
| Head length (jaw) | -0.98390 | -0.09005 | -0.00753 | 0.08912 | -0.03961 |
| Head width | -0.96179 | -0.22482 | -0.10203 | 0.08607 | -0.00690 |
| Eye-naris distance | -0.96443 | -0.06946 | -0.10840 | -0.00954 | 0.02618 |
| Interorbital span | -0.90824 | -0.23444 | -0.23177 | -0.02446 | 0.00251 |
| Internarial span | -0.82743 | -0.23964 | -0.30424 | 0.08800 | -0.10378 |
| Naris-Snout distance | -0.88144 | -0.01217 | -0.07706 | 0.09741 | -0.06098 |
| Eye length | -0.93872 | -0.11881 | -0.15223 | -0.03578 | -0.08032 |
| Mouth width | -0.95130 | -0.23767 | -0.11446 | 0.14250 | 0.02409 |
| Humerus length | -0.98189 | -0.11588 | -0.04530 | -0.04193 | -0.02147 |
| Forearm length | -0.97657 | -0.07363 | -0.06071 | -0.06249 | -0.01949 |
| Wrist width | -0.96895 | -0.13045 | -0.00827 | -0.06064 | -0.04309 |
| Hand length | -0.98084 | -0.05615 | -0.07871 | -0.01190 | 0.12152 |
| Thumb length | -0.97318 | -0.03892 | 0.04930 | 0.03591 | 0.05340 |
| Finger 4 length | -0.95684 | 0.07443 | -0.00168 | -0.01185 | 0.21834 |
| Femur length | -0.98432 | 0.11702 | -0.06435 | -0.05228 | -0.06797 |
| Femur width | -0.95332 | -0.08968 | 0.06907 | 0.08767 | -0.12861 |
| Tibial length | -0.97526 | 0.19121 | -0.07807 | -0.01902 | -0.04054 |
| Tibial width | -0.96955 | -0.02436 | 0.00393 | -0.05311 | -0.15050 |
| Foot length (toe 1) | -0.97856 | 0.18182 | 0.04311 | 0.02471 | 0.02170 |
| Foot length (total) | -0.98673 | 0.14917 | -0.02569 | -0.00086 | 0.03345 |
| Toe 1 length | -0.96046 | 0.12200 | 0.08229 | 0.12548 | 0.08098 |
| Toe 5 length | -0.94568 | 0.23988 | 0.11874 | 0.13939 | 0.02479 |
| Webbing 4-5 length | -0.84145 | 0.27201 | 0.24578 | 0.31528 | -0.11150 |
