# Supplementary material for: Invasive cane toads are unique in shape but overlap in ecological niche compared to Australian native frogs
Source: Ecol Evol. 2017 Aug 17;7(19):7609–19. doi: 10.1002/ece3.3253 (PMC5632638; doi:10.1002/ece3.3253)
Supplement: Supplementary file 10 [file ECE3-7-7609-s010.docx]

| Table S4. Phylogenetic PCA on phylogenetically size-corrected data, using *phytools*. | | | | | |
| --- | --- | --- | --- | --- | --- |
|  |  |  |  |  |  |
| **Axis** | **1** | **2** | **3** | **4** | **5** |
| **Eigenvalues (λ)** | **769.94730** | **190.64410** | **97.39574** | **51.57547** | **43.55859** |
| **Total variance explained (%)** | **59.43965** | **14.71765** | **7.51892** | **3.98161** | **3.36271** |
| **SVL** | 0.00600 | -0.03629 | -0.01363 | 0.02095 | -0.01692 |
| Head length (jaw) | -0.21396 | 0.51499 | 0.49612 | 0.21634 | 0.22837 |
| Head width | 0.24804 | 0.87054 | 0.27029 | 0.02209 | -0.08686 |
| Eye-naris distance | -0.23666 | 0.52797 | -0.12103 | -0.09578 | -0.12912 |
| Interorbital span | 0.15545 | 0.73580 | -0.20182 | -0.02318 | -0.45842 |
| Internarial span | 0.08844 | 0.69904 | -0.01525 | 0.17419 | -0.19485 |
| Naris-Snout distance | -0.22677 | 0.26502 | 0.14621 | 0.12952 | 0.35302 |
| Eye length | -0.08846 | 0.58379 | -0.18492 | 0.21247 | 0.08550 |
| Mouth width | 0.21945 | 0.85776 | 0.39301 | -0.07610 | 0.11277 |
| Humerus length | -0.09626 | 0.62288 | -0.22089 | 0.10950 | 0.07631 |
| Forearm length | -0.24785 | 0.47161 | -0.27800 | 0.07426 | 0.19067 |
| Wrist width | 0.06324 | 0.30084 | -0.16422 | 0.17929 | -0.09716 |
| Hand length | -0.34949 | 0.54691 | -0.10177 | -0.55019 | -0.34365 |
| Thumb length | -0.25610 | 0.05309 | 0.27974 | -0.22900 | -0.16382 |
| Finger 4 length | -0.54967 | 0.02616 | -0.01916 | -0.64994 | -0.28214 |
| Femur length | -0.87319 | 0.18197 | -0.25510 | 0.24219 | -0.14904 |
| Femur width | -0.10163 | 0.11065 | 0.38644 | 0.44412 | 0.21382 |
| Tibial length | -0.95047 | 0.13464 | -0.14150 | 0.11861 | -0.11025 |
| Tibial width | -0.34382 | 0.11481 | -0.14172 | 0.57597 | 0.19304 |
| Foot length (toe 1) | -0.93871 | -0.15582 | 0.12661 | -0.06708 | 0.18363 |
| Foot length (total) | -0.97433 | 0.06734 | -0.02583 | -0.11930 | 0.14749 |
| Toe 1 length | -0.66765 | -0.11683 | 0.43219 | -0.23599 | -0.13590 |
| Toe 5 length | -0.80994 | -0.27570 | 0.42038 | -0.06248 | -0.19585 |
| Webbing 4-5 length | -0.55569 | -0.33080 | 0.64195 | 0.18548 | -0.27413 |
