# Supplementary material for: Invasive cane toads are unique in shape but overlap in ecological niche compared to Australian native frogs
Source: Ecol Evol. 2017 Aug 17;7(19):7609–19. doi: 10.1002/ece3.3253 (PMC5632638; doi:10.1002/ece3.3253)
Supplement: Supplementary file 11 [file ECE3-7-7609-s011.docx]

| Table S5. Dunnett's multiple comparison test for selected morphological and environmental variables. Rhinella marina (R.) was tested against each Australian frog clade. | | | | | | | | | | | | | | | | | | | | | | | | | | | | |
| --- | --- | --- | --- | --- | --- | --- | --- | --- | --- | --- | --- | --- | --- | --- | --- | --- | --- | --- | --- | --- | --- | --- | --- | --- | --- | --- | --- | --- |
|  |  |  |  |  |  |  |  |  |  |  |  |  |  |  |  |  |  |  |  |  |  |  |  |  |  |  |  |  |
| **Comparisons** | **SVL** | | | | **RLLR (arm l. / leg l.)** | | | | **Morphological PC 1sc** | | | | **Morphological PC 2sc** | | | | **Morphological PC 3sc** | | | | **PC 1 Environment** | | | | **PC 2 Environment** | | | |
|  | **Est.** | **SE** | **t** | **p** | **Est.** | **SE** | **t** | **p** | **Est.** | **SE** | **t** | **p** | **Est.** | **SE** | **t** | **p** | **Est.** | **SE** | **t** | **p** | **Est.** | **SE** | **t** | **p** | **Est.** | **SE** | **t** | **p** |
| *R. - Austrochaperina* | **-105.34** | **2.52** | **-41.77** | **< 0.001** | **-0.14** | **0.01** | **-16.71** | **< 0.001** | **-13.77** | **0.95** | **-14.50** | **<0.001** | 0.15 | 0.53 | 0.29 | 1.00 | -1.13 | 0.54 | -2.10 | 0.69 | 0.13 | 0.08 | 1.57 | 0.99 | **1.69** | **0.05** | **30.89** | **<0.001** |
| *R. - Cophixalus* | **-104.15** | **1.85** | **-56.43** | **< 0.001** | **-0.09** | **0.01** | **-14.01** | **< 0.001** | **-14.08** | **0.69** | **-20.26** | **<0.001** | -0.70 | 0.39 | -1.79 | 0.91 | -1.37 | 0.39 | -3.47 | 0.02 | **1.62** | **0.09** | **17.68** | **<0.001** | **2.02** | **0.06** | **33.09** | **<0.001** |
| *R. - Rana* | **-41.72** | **4.49** | **-9.30** | **< 0.001** | **-0.18** | **0.01** | **-12.09** | **< 0.001** | **-43.38** | **1.69** | **-25.68** | **<0.001** | **8.65** | **0.95** | **9.15** | **< 0.001** | **-7.64** | **0.96** | **-7.98** | **< 0.001** | **-0.87** | **0.25** | **-3.54** | **0.02** | **1.68** | **0.16** | **10.20** | **<0.001** |
| *R. - Cyclorana* | **-78.83** | **1.88** | **-41.88** | **< 0.001** | **-0.08** | **0.01** | **-12.29** | **< 0.001** | **-11.55** | **0.71** | **-16.30** | **<0.001** | **-2.27** | **0.40** | **-5.71** | **< 0.001** | **-3.60** | **0.40** | **-8.96** | **< 0.001** | **-3.90** | **0.04** | **-88.02** | **<0.001** | **-1.26** | **0.03** | **-42.74** | **<0.001** |
| R. - Litoria 1 | **-101.66** | **2.73** | **-37.25** | **< 0.001** | **-0.20** | **0.01** | **-22.42** | **< 0.001** | **-16.38** | **1.03** | **-15.94** | **<0.001** | **4.28** | **0.58** | **7.44** | **< 0.001** | **-2.40** | **0.58** | **-4.13** | **0.00** | **0.59** | **0.04** | **15.83** | **<0.001** | **-0.81** | **0.02** | **-32.40** | **<0.001** |
| R. - Litoria 2 | **-84.15** | **4.87** | **-17.27** | **< 0.001** | **-0.16** | **0.02** | **-9.95** | **< 0.001** | **-18.13** | **1.84** | **-9.88** | **<0.001** | **6.32** | **1.03** | **6.15** | **< 0.001** | -1.65 | 1.04 | -1.59 | 0.98 | **-0.56** | **0.12** | **-4.53** | **<0.001** | **-2.78** | **0.08** | **-33.51** | **<0.001** |
| R. - Litoria 3 | **-40.19** | **4.87** | **-8.25** | **< 0.001** | **-0.14** | **0.02** | **-8.57** | **< 0.001** | **-36.33** | **1.84** | **-19.80** | **<0.001** | -0.38 | 1.03 | -0.37 | 1.00 | -2.48 | 1.04 | -2.38 | 0.44 | -0.33 | 0.85 | -0.38 | 1.00 | 0.23 | 0.57 | 0.41 | 1.00 |
| R. - Litoria 4 | **-51.65** | **2.90** | **-17.81** | **< 0.001** | **-0.17** | **0.01** | **-17.85** | **< 0.001** | **-26.35** | **1.09** | **-24.13** | **<0.001** | **6.20** | **0.61** | **10.14** | **< 0.001** | -0.48 | 0.62 | -0.78 | 1.00 | **0.45** | **0.05** | **9.88** | **<0.001** | **-3.11** | **0.03** | **-103.38** | **<0.001** |
| R. - Litoria 5 | **-81.97** | **2.56** | **-32.05** | **< 0.001** | **-0.20** | **0.01** | **-23.78** | **< 0.001** | **-28.23** | **0.96** | **-29.31** | **<0.001** | 0.88 | 0.54 | 1.64 | 0.97 | **1.92** | **0.55** | **3.52** | **0.02** | **1.03** | **0.04** | **27.57** | **<0.001** | **-1.01** | **0.02** | **-40.42** | **<0.001** |
| R. - Litoria 6 | **-86.00** | **4.87** | **-17.65** | **< 0.001** | **-0.11** | **0.02** | **-6.57** | **< 0.001** | **-14.77** | **1.84** | **-8.05** | **<0.001** | 3.04 | 1.03 | 2.96 | 0.11 | -3.40 | 1.04 | -3.27 | 0.04 | **1.39** | **0.14** | **10.00** | **<0.001** | **-0.92** | **0.09** | **-9.98** | **<0.001** |
| R. - Litoria 7 | **-37.76** | **4.87** | **-7.75** | **< 0.001** | **-0.19** | **0.02** | **-11.71** | **< 0.001** | **-30.26** | **1.84** | **-16.49** | **<0.001** | **6.84** | **1.03** | **6.65** | **< 0.001** | 2.36 | 1.04 | 2.27 | 0.54 | -0.27 | 0.18 | -1.50 | 1.00 | **1.65** | **0.12** | **13.83** | **<0.001** |
| R. - Litoria 8 | **-52.26** | **2.68** | **-19.49** | **< 0.001** | **-0.09** | **0.01** | **-10.70** | **< 0.001** | **-16.56** | **1.01** | **-16.40** | **<0.001** | **5.40** | **0.57** | **9.54** | **< 0.001** | **-4.83** | **0.57** | **-8.43** | **< 0.001** | **-1.04** | **0.04** | **-26.51** | **<0.001** | **-1.12** | **0.03** | **-42.70** | **<0.001** |
| R. - Litoria 9 | **-74.28** | **3.04** | **-24.41** | **< 0.001** | **-0.14** | **0.01** | **-14.45** | **< 0.001** | **-21.19** | **1.15** | **-18.49** | **<0.001** | **3.46** | **0.64** | **5.39** | **< 0.001** | -1.29 | 0.65 | -1.99 | 0.78 | **1.37** | **0.06** | **22.52** | **<0.001** | **-0.20** | **0.04** | **-4.89** | **<0.001** |
| R. - Litoria 10 | **-77.69** | **4.87** | **-15.94** | **< 0.001** | **-0.18** | **0.02** | **-11.15** | **< 0.001** | **-25.60** | **1.84** | **-13.95** | **<0.001** | **4.04** | **1.03** | **3.93** | **0.00** | -0.49 | 1.04 | -0.48 | 1.00 | **-2.47** | **0.16** | **-15.11** | **<0.001** | **0.61** | **0.11** | **5.59** | **<0.001** |
| R. - Litoria 11 | **-71.69** | **3.04** | **-23.56** | **< 0.001** | **-0.16** | **0.01** | **-15.56** | **< 0.001** | **-25.36** | **1.15** | **-22.14** | **<0.001** | **3.65** | **0.64** | **5.69** | **< 0.001** | -0.06 | 0.65 | -0.09 | 1.00 | **0.87** | **0.16** | **5.34** | **<0.001** | **1.08** | **0.11** | **9.93** | **<0.001** |
| R. - Litoria 12 | **-92.05** | **2.21** | **-41.70** | **< 0.001** | **-0.14** | **0.01** | **-19.26** | **< 0.001** | **-19.22** | **0.83** | **-23.12** | **<0.001** | **2.75** | **0.47** | **5.91** | **< 0.001** | **-1.80** | **0.47** | **-3.83** | **0.01** | **1.54** | **0.03** | **45.47** | **<0.001** | **-3.03** | **0.02** | **-134.18** | **<0.001** |
| R. - Litoria 13 | **-92.19** | **1.96** | **-47.14** | **< 0.001** | **-0.20** | **0.01** | **-31.13** | **< 0.001** | **-23.76** | **0.74** | **-32.27** | **<0.001** | **2.08** | **0.41** | **5.04** | **< 0.001** | -0.75 | 0.42 | -1.79 | 0.91 | **-0.87** | **0.03** | **-28.03** | **<0.001** | **-0.61** | **0.02** | **-29.29** | **<0.001** |
| R. - Litoria 14 | **-108.26** | **2.90** | **-37.34** | **< 0.001** | **-0.19** | **0.01** | **-19.51** | **< 0.001** | **-18.07** | **1.09** | **-16.55** | **<0.001** | **2.44** | **0.61** | **3.99** | **0.00** | -2.71 | 0.62 | -4.38 | < 0.001 | **-2.41** | **0.09** | **-27.08** | **<0.001** | **0.85** | **0.06** | **14.29** | **<0.001** |
| R. - Litoria 15 | **-49.74** | **3.59** | **-13.86** | **< 0.001** | **-0.13** | **0.01** | **-11.04** | **< 0.001** | **-24.04** | **1.35** | **-17.79** | **<0.001** | **7.07** | **0.76** | **9.34** | **< 0.001** | **-2.66** | **0.77** | **-3.47** | **0.02** | **-0.99** | **0.10** | **-9.72** | **<0.001** | **-2.87** | **0.07** | **-42.35** | **<0.001** |
| R. - Litoria 16 | **-88.51** | **2.73** | **-32.43** | **< 0.001** | **-0.15** | **0.01** | **-16.38** | **< 0.001** | **-23.40** | **1.03** | **-22.77** | **<0.001** | 1.19 | 0.58 | 2.07 | 0.72 | -1.32 | 0.58 | -2.27 | 0.54 | **1.06** | **0.11** | **9.85** | **<0.001** | **1.60** | **0.07** | **22.29** | **<0.001** |
| R. - Litoria 17 | **-80.35** | **3.04** | **-26.40** | **< 0.001** | **-0.14** | **0.01** | **-14.26** | **< 0.001** | **-22.13** | **1.15** | **-19.32** | **<0.001** | **4.42** | **0.64** | **6.89** | **< 0.001** | -0.57 | 0.65 | -0.87 | 1.00 | 0.06 | 0.03 | 1.69 | 0.97 | **-1.72** | **0.02** | **-77.97** | **<0.001** |
| R. - Litoria 18 | **-86.32** | **2.13** | **-40.51** | **< 0.001** | **-0.14** | **0.01** | **-19.71** | **< 0.001** | **-21.20** | **0.80** | **-26.43** | **<0.001** | **1.98** | **0.45** | **4.40** | **< 0.001** | **-1.86** | **0.45** | **-4.08** | **0.00** | **2.01** | **0.04** | **49.27** | **<0.001** | **-1.45** | **0.03** | **-53.01** | **<0.001** |
| R. - Litoria 19 | **-88.50** | **3.04** | **-29.08** | **< 0.001** | **-0.08** | **0.01** | **-8.32** | **< 0.001** | **-10.25** | **1.15** | **-8.95** | **<0.001** | **7.42** | **0.64** | **11.56** | **< 0.001** | **-3.69** | **0.65** | **-5.68** | **< 0.001** | **-1.65** | **0.04** | **-43.76** | **<0.001** | **-1.43** | **0.03** | **-56.98** | **<0.001** |
| R. - Litoria 20 | **-84.28** | **6.18** | **-13.63** | **< 0.001** | **-0.11** | **0.02** | **-5.32** | **< 0.001** | **-23.03** | **2.33** | **-9.89** | **<0.001** | 2.77 | 1.30 | 2.12 | 0.67 | -1.53 | 1.32 | -1.16 | 1.00 | **5.27** | **0.17** | **31.11** | **<0.001** | **-2.94** | **0.11** | **-26.07** | **<0.001** |
| *R. - Adelotus* | **-96.20** | **4.87** | **-19.74** | **< 0.001** | **-0.12** | **0.02** | **-7.73** | **< 0.001** | **-14.94** | **1.84** | **-8.14** | **<0.001** | -0.38 | 1.03 | -0.37 | 1.00 | -1.27 | 1.04 | -1.22 | 1.00 | **1.73** | **0.06** | **28.24** | **<0.001** | **-0.87** | **0.04** | **-21.32** | **<0.001** |
| *R. - Arenohpryne* | **-101.75** | **3.59** | **-28.35** | **< 0.001** | **0.25** | **0.01** | **21.38** | **< 0.001** | -3.94 | 1.35 | -2.92 | 0.13 | 0.40 | 0.76 | 0.53 | 1.00 | **-5.20** | **0.77** | **-6.78** | **< 0.001** | **-4.37** | **0.39** | **-11.22** | **<0.001** | **-2.78** | **0.26** | **-10.69** | **<0.001** |
| *R. - Assa* | **-108.36** | **4.87** | **-22.23** | **< 0.001** | **-0.09** | **0.02** | **-5.51** | **< 0.001** | **-12.20** | **1.84** | **-6.65** | **<0.001** | -1.36 | 1.03 | -1.33 | 1.00 | -3.09 | 1.04 | -2.97 | 0.11 | **3.23** | **0.11** | **30.53** | **<0.001** | **0.38** | **0.07** | **5.45** | **<0.001** |
| *R. - Crinia* | **-107.83** | **1.85** | **-58.27** | **< 0.001** | **-0.11** | **0.01** | **-17.66** | **< 0.001** | **-14.57** | **0.70** | **-20.92** | **<0.001** | 0.47 | 0.39 | 1.21 | 1.00 | -0.96 | 0.39 | -2.43 | 0.40 | **0.83** | **0.03** | **30.87** | **<0.001** | **-2.51** | **0.02** | **-139.67** | **<0.001** |
| *R. - Geocrinia* | **-104.52** | **2.26** | **-46.20** | **< 0.001** | **-0.07** | **0.01** | **-9.78** | **< 0.001** | **-13.48** | **0.85** | **-15.83** | **<0.001** | -0.59 | 0.48 | -1.24 | 1.00 | -0.84 | 0.48 | -1.73 | 0.94 | **2.17** | **0.07** | **32.30** | **<0.001** | **-3.18** | **0.04** | **-70.96** | **<0.001** |
| *R. - Heleioporus* | **-68.44** | **2.40** | **-28.51** | **< 0.001** | **0.05** | **0.01** | **6.60** | **< 0.001** | **-7.48** | **0.90** | **-8.28** | **<0.001** | **-6.73** | **0.51** | **-13.29** | **< 0.001** | **-3.81** | **0.51** | **-7.43** | **< 0.001** | -0.05 | 0.06 | -0.87 | 1.00 | **-2.44** | **0.04** | **-64.23** | **<0.001** |
| *R. - Lechriodus* | **-77.82** | **4.87** | **-15.97** | **< 0.001** | **-0.15** | **0.02** | **-9.22** | **< 0.001** | **-27.27** | **1.84** | **-14.86** | **<0.001** | **-3.36** | **1.03** | **-3.27** | **0.04** | **5.02** | **1.04** | **4.83** | **< 0.001** | **2.41** | **0.09** | **27.05** | **<0.001** | **-0.55** | **0.06** | **-9.29** | **<0.001** |
| *R. - Limnodynastes* | **-72.84** | **2.00** | **-36.47** | **< 0.001** | **-0.06** | **0.01** | **-9.24** | **< 0.001** | **-12.87** | **0.75** | **-17.12** | **<0.001** | **-3.38** | **0.42** | **-8.02** | **< 0.001** | -0.86 | 0.43 | -2.03 | 0.76 | **-0.30** | **0.03** | **-11.15** | **<0.001** | **-2.50** | **0.02** | **-138.30** | **<0.001** |
| *R. - Metacrinia* | **-107.11** | **4.87** | **-21.98** | **< 0.001** | **0.08** | **0.02** | **5.05** | **< 0.001** | **-9.16** | **1.84** | **-4.99** | **<0.001** | **3.96** | **1.03** | **3.85** | **0.01** | **-10.14** | **1.04** | **-9.75** | **< 0.001** | 0.02 | 0.26 | 0.08 | 1.00 | **-2.51** | **0.18** | **-14.34** | **<0.001** |
| *R. - Mixophyes* | **-53.01** | **2.18** | **-24.38** | **< 0.001** | **-0.19** | **0.01** | **-26.17** | **< 0.001** | **-46.25** | **0.82** | **-56.47** | **<0.001** | **-3.81** | **0.46** | **-8.31** | **< 0.001** | **-7.73** | **0.46** | **-16.65** | **< 0.001** | **1.47** | **0.05** | **29.40** | **<0.001** | **-0.41** | **0.03** | **-12.14** | **<0.001** |
| *R. - Myobatrachus* | **-73.44** | **4.49** | **-16.37** | **< 0.001** | **0.29** | **0.01** | **19.32** | **< 0.001** | **9.09** | **1.69** | **5.38** | **<0.001** | **9.56** | **0.95** | **10.11** | **< 0.001** | **-5.38** | **0.96** | **-5.62** | **< 0.001** | **-2.39** | **0.15** | **-15.87** | **<0.001** | **-2.57** | **0.10** | **-25.57** | **<0.001** |
| *R. - Neobatrachus* | **-79.67** | **2.08** | **-38.31** | **< 0.001** | **0.03** | **0.01** | **3.90** | **0.00** | **-9.09** | **0.78** | **-11.61** | **<0.001** | **-5.05** | **0.44** | **-11.50** | **< 0.001** | **-4.70** | **0.44** | **-10.60** | **< 0.001** | **-3.10** | **0.04** | **-70.98** | **<0.001** | **-3.23** | **0.03** | **-110.93** | **<0.001** |
| *R. - Notaden* | **-74.97** | **2.73** | **-27.47** | **< 0.001** | **0.05** | **0.01** | **5.84** | **< 0.001** | -2.39 | 1.03 | -2.33 | 0.49 | **3.36** | **0.58** | **5.84** | **< 0.001** | **-2.40** | **0.58** | **-4.12** | **0.00** | **-4.32** | **0.07** | **-60.13** | **<0.001** | **-0.84** | **0.05** | **-17.53** | **<0.001** |
| *R. - Paracrinia* | **-97.73** | **4.87** | **-20.05** | **< 0.001** | **-0.08** | **0.02** | **-5.20** | **< 0.001** | **-13.79** | **1.84** | **-7.51** | **<0.001** | -0.64 | 1.03 | -0.63 | 1.00 | 0.07 | 1.04 | 0.07 | 1.00 | **1.67** | **0.11** | **15.15** | **<0.001** | **-1.96** | **0.07** | **-26.61** | **<0.001** |
| *R. - Philoria* | **-97.89** | **2.37** | **-41.23** | **< 0.001** | **-0.08** | **0.01** | **-10.09** | **< 0.001** | **-13.40** | **0.89** | **-14.99** | **<0.001** | -0.50 | 0.50 | -1.01 | 1.00 | -1.30 | 0.51 | -2.56 | 0.31 | **2.27** | **0.10** | **21.79** | **<0.001** | -0.14 | 0.07 | -2.06 | 0.78 |
| *R. - Platyplectrum* | **-88.35** | **3.59** | **-24.61** | **< 0.001** | **-0.05** | **0.01** | **-4.35** | **< 0.001** | **-12.31** | **1.35** | **-9.11** | **<0.001** | -0.38 | 0.76 | -0.50 | 1.00 | -2.14 | 0.77 | -2.80 | 0.17 | **-1.33** | **0.06** | **-21.98** | **<0.001** | **-1.23** | **0.04** | **-30.43** | **<0.001** |
| *R. - Pseudophryne* | **-101.47** | **1.89** | **-53.73** | **< 0.001** | **0.04** | **0.01** | **6.10** | **< 0.001** | **-8.23** | **0.71** | **-11.57** | **<0.001** | **2.16** | **0.40** | **5.42** | **< 0.001** | **-2.18** | **0.40** | **-5.41** | **< 0.001** | **0.88** | **0.03** | **26.45** | **<0.001** | **-1.78** | **0.02** | **-80.49** | **<0.001** |
| *R. - Rheobatrachus* | **-76.79** | **3.59** | **-21.39** | **< 0.001** | **-0.08** | **0.01** | **-6.69** | **< 0.001** | **-14.19** | **1.35** | **-10.50** | **<0.001** | 0.99 | 0.76 | 1.31 | 1.00 | **-3.19** | **0.77** | **-4.17** | **0.00** | **1.62** | **0.49** | **3.30** | **0.04** | 0.51 | 0.33 | 1.56 | 0.99 |
| *R. - Spicospina* | **-96.86** | **4.87** | **-19.87** | **< 0.001** | 0.03 | 0.02 | 1.64 | 0.97 | **-8.72** | **1.84** | **-4.75** | **<0.001** | 0.01 | 1.03 | 0.01 | 1.00 | -2.59 | 1.04 | -2.49 | 0.36 | 0.28 | 1.10 | 0.25 | 1.00 | **-2.49** | **0.73** | **-3.40** | **0.03** |
| *R. - Taudactylus* | **-101.44** | **2.37** | **-42.73** | **< 0.001** | **-0.16** | **0.01** | **-20.35** | **< 0.001** | **-16.95** | **0.89** | **-18.96** | **<0.001** | 0.11 | 0.50 | 0.21 | 1.00 | -0.03 | 0.51 | -0.06 | 1.00 | **1.21** | **0.22** | **5.47** | **<0.001** | **1.47** | **0.15** | **9.93** | **<0.001** |
| *R. - Uperoleia* | **-103.94** | **1.70** | **-61.22** | **< 0.001** | 0.00 | 0.01 | 0.38 | 1.00 | **-11.23** | **0.64** | **-17.57** | **<0.001** | -0.11 | 0.36 | -0.31 | 1.00 | **-2.18** | **0.36** | **-6.01** | **< 0.001** | **-1.21** | **0.04** | **-31.42** | **<0.001** | **-0.45** | **0.03** | **-17.42** | **<0.001** |
