# Supplementary material for: Invasive cane toads are unique in shape but overlap in ecological niche compared to Australian native frogs
Source: Ecol Evol. 2017 Aug 17;7(19):7609–19. doi: 10.1002/ece3.3253 (PMC5632638; doi:10.1002/ece3.3253)
Supplement: Supplementary file 12 [file ECE3-7-7609-s012.docx]

| Table S6. Bartlett's test of homogeneity of variances per clade in SVL, RLLR, and each of the size-corrected variables (residuals of the linear regression of morphological raw variables against SVL) | | | |
| --- | --- | --- | --- |
| **Variable** | **Bartlett's K-squared** | **d.f.** | **p-value** |
| **SVL** | 884.6458 | 45 | < 0.001 |
| **RLLR** | 244.1918 | 45 | < 0.001 |
| Head length (jaw) - residuals | 665.655 | 45 | < 0.001 |
| Head width - residuals | 660.8761 | 45 | < 0.001 |
| Eye-naris distance - residuals | 777.4883 | 45 | < 0.001 |
| Interorbital span - residuals | 643.0143 | 45 | < 0.001 |
| Internarial span - residuals | 435.8912 | 45 | < 0.001 |
| Naris-Snout distance - residuals | 641.9193 | 45 | < 0.001 |
| Eye length - residuals | 617.4222 | 45 | < 0.001 |
| Mouth width - residuals | 509.0844 | 45 | < 0.001 |
| Humerus length - residuals | 479.6224 | 45 | < 0.001 |
| Forearm length - residuals | 608.6104 | 45 | < 0.001 |
| Wrist width - residuals | 525.5688 | 45 | < 0.001 |
| Hand length - residuals | 424.1632 | 45 | < 0.001 |
| Thumb length - residuals | 632.5388 | 45 | < 0.001 |
| Finger 4 length - residuals | 694.2849 | 45 | < 0.001 |
| Femur length - residuals | 444.5215 | 45 | < 0.001 |
| Femur width - residuals | 663.3449 | 45 | < 0.001 |
| Tibial length - residuals | 714.862 | 45 | < 0.001 |
| Tibial width - residuals | 551.7479 | 45 | < 0.001 |
| Foot length (toe 1) - residuals | 429.7523 | 45 | < 0.001 |
| Foot length (total) - residuals | 447.423 | 45 | < 0.001 |
| Toe 1 length - residuals | 372.668 | 45 | < 0.001 |
| Toe 5 length - residuals | 871.1247 | 45 | < 0.001 |
| Webbing 4-5 length - residuals | 1489.029 | 45 | < 0.001 |
| Elbow-axilla - residuals | 590.1461 | 45 | < 0.001 |
