# Supplementary material for: Invasive cane toads are unique in shape but overlap in ecological niche compared to Australian native frogs
Source: Ecol Evol. 2017 Aug 17;7(19):7609–19. doi: 10.1002/ece3.3253 (PMC5632638; doi:10.1002/ece3.3253)
Supplement: Supplementary file 13 [file ECE3-7-7609-s013.docx]

| Table S7. F-test one-way analysis of variance for selected morphological and environmental variables. *Rhinella marina* (R.) was tested against each Australian frog clade. Results are depicted in bold when *Rhinella marina*'s variance was significatively higher. | | | | | | | | | | | | | | |
| --- | --- | --- | --- | --- | --- | --- | --- | --- | --- | --- | --- | --- | --- | --- |
|  |  |  |  |  |  |  |  |  |  |  |  |  |  |  |
| **Comparisons** | **SVL** | | **RLLR (arm l. / leg l.)** | | **Morphol PC 1sc** | | **Morpho PC 2sc** | | **Morpho PC 3sc** | | **PC 1 Env** | | **PC 2 Env** | |
|  | **F** | **p** | **F** | **p** | **F** | **p** | **F** | **p** | **F** | **p** | **F** | **p** | **F** | **p** |
| *R. - Austrochaperina* | **22.502** | **> 0.001** | 0.987 | 0.935 | 0.542 | 0.065 | 0.841 | 0.588 | 1.326 | 0.455 | **1.232** | **> 0.001** | **1.334** | **> 0.001** |
| *R. - Cophixalus* | **5.778** | **>0.001** | 0.264 | >0.001 | 0.201 | >0.001 | 0.284 | >0.001 | 0.143 | >0.001 | **2.138** | **>0.001** | 0.905 | 0.131 |
| *R. - Rana* | **143.075** | **>0.001** | 5.557 | 0.062 | **7.758** | **0.030** | 4.642 | 0.091 | 5.401 | 0.066 | **3.213** | **>0.001** | **1.844** | **0.003** |
| *R. - Cyclorana* | **2.728** | **>0.001** | 0.409 | >0.001 | 0.397 | >0.001 | 0.374 | >0.001 | 0.421 | 0.001 | **1.816** | **>0.001** | 0.232 | >0.001 |
| R. - Litoria 1 | **101.448** | **>0.001** | 0.629 | 0.187 | 0.206 | >0.001 | 0.345 | 0.002 | 0.332 | 0.002 | **1.468** | **>0.001** | 0.426 | >0.001 |
| R. - Litoria 2 | **143.365** | **>0.001** | 1.341 | 0.869 | 0.504 | 0.220 | 0.611 | 0.357 | 0.904 | 0.726 | **3.184** | **>0.001** | **6.004** | **>0.001** |
| R. - Litoria 3 | 2.402 | 0.408 | 0.714 | 0.492 | 1.664 | 0.673 | 1.046 | 0.878 | 1.288 | 0.909 | 2.069 | 0.504 | 0.280 | 0.013 |
| R. - Litoria 4 | **8.382** | **>0.001** | 0.114 | >0.001 | 0.476 | 0.045 | 0.435 | 0.024 | 1.062 | 0.940 | **1.418** | **>0.001** | 0.564 | >0.001 |
| R. - Litoria 5 | **6.308** | **>0.001** | 0.388 | 0.005 | 0.254 | >0.001 | 0.760 | 0.405 | 0.316 | >0.001 | **1.433** | **>0.001** | 0.256 | >0.001 |
| R. - Litoria 6 | **100.328** | **>0.001** | 2.844 | 0.317 | 1.288 | 0.909 | 2.238 | 0.452 | 1.315 | 0.888 | **4.196** | **>0.001** | **2.867** | **>0.001** |
| R. - Litoria 7 | 1.037 | 0.870 | 0.079 | >0.001 | 0.938 | 0.765 | 0.313 | 0.040 | 0.809 | 0.614 | **1.715** | **>0.001** | 0.614 | >0.001 |
| R. - Litoria 8 | 1.095 | 0.854 | 0.791 | 0.487 | 0.875 | 0.677 | 0.566 | 0.102 | 0.412 | 0.011 | 0.709 | >0.001 | 0.230 | >0.001 |
| R. - Litoria 9 | **3.066** | **0.025** | 1.091 | 0.906 | 0.494 | 0.067 | 1.028 | 0.985 | 0.603 | 0.188 | **2.480** | **>0.001** | 0.471 | >0.001 |
| R. - Litoria 10 | 7.986 | 0.055 | 3.138 | 0.272 | 0.684 | 0.453 | 5.272 | 0.115 | 1.718 | 0.646 | **6.741** | **>0.001** | **2.353** | **>0.001** |
| R. - Litoria 11 | **2.719** | **0.043** | 0.772 | 0.484 | **3.198** | **0.020** | 0.506 | 0.077 | 0.707 | 0.358 | **2.385** | **>0.001** | 0.272 | >0.001 |
| R. - Litoria 12 | **11.796** | **>0.001** | 1.064 | 0.853 | 0.262 | >0.001 | 0.624 | 0.114 | 0.394 | 0.002 | **1.588** | **>0.001** | 0.686 | >0.001 |
| R. - Litoria 13 | **8.184** | **>0.001** | 0.528 | 0.019 | 0.363 | >0.001 | 0.581 | 0.046 | 0.206 | >0.001 | 0.882 | >0.001 | 0.248 | >0.001 |
| R. - Litoria 14 | **35.104** | **>0.001** | 0.743 | 0.410 | 0.331 | 0.003 | 0.449 | 0.030 | 0.276 | >0.001 | **4.653** | **>0.001** | **2.202** | **>0.001** |
| R. - Litoria 15 | **19.431** | **>0.001** | 0.829 | 0.622 | 0.845 | 0.649 | 0.442 | 0.064 | 1.004 | 0.909 | **3.245** | **>0.001** | **1.307** | **>0.001** |
| R. - Litoria 16 | **6.720** | **>0.001** | 1.814 | 0.154 | 1.172 | 0.725 | 0.545 | 0.085 | 0.286 | >0.001 | **3.124** | **>0.001** | 0.438 | >0.001 |
| R. - Litoria 17 | **28.712** | **>0.001** | 0.736 | 0.413 | 0.660 | 0.275 | 0.662 | 0.279 | 0.863 | 0.667 | 0.979 | 0.382 | 0.250 | >0.001 |
| R. - Litoria 18 | **3.564** | **>0.001** | 1.204 | 0.536 | 0.694 | 0.207 | 1.000 | 0.991 | 1.174 | 0.593 | **7.389** | **>0.001** | 0.796 | >0.001 |
| R. - Litoria 19 | **31.932** | **>0.001** | 0.409 | 0.020 | 0.103 | >0.001 | 1.537 | 0.380 | 0.396 | 0.015 | 0.546 | >0.001 | 0.237 | >0.001 |
| R. - Litoria 20 | 9.372 | 0.202 | 1.649 | 0.902 | 0.248 | 0.047 | 0.280 | 0.070 | 0.189 | 0.016 | **10.139** | **>0.001** | **11.430** | **>0.001** |
| *R. - Adelotus* | **160.896** | **>0.001** | 0.875 | 0.694 | 2.020 | 0.521 | 1.204 | 0.976 | 1.548 | 0.736 | **6.418** | **>0.001** | **1.251** | **>0.001** |
| *R. - Arenohpryne* | **131.715** | **>0.001** | 0.079 | >0.001 | 1.246 | 0.769 | 0.353 | 0.017 | 1.274 | 0.738 | **34.079** | **>0.001** | **64.208** | **>0.001** |
| *R. - Assa* | **1968.670** | **>0.001** | 1.455 | 0.792 | **9.059** | **0.043** | **10.385** | **0.034** | **9.280** | **0.041** | **7.894** | **>0.001** | **1.625** | **>0.001** |
| *R. - Crinia* | **34.568** | **>0.001** | 0.398 | >0.001 | 0.358 | >0.001 | 0.397 | >0.001 | 0.495 | 0.008 | **1.215** | **>0.001** | 0.395 | >0.001 |
| *R. - Geocrinia* | **43.002** | **>0.001** | 0.231 | >0.001 | 0.325 | >0.001 | 0.321 | >0.001 | 0.304 | >0.001 | **2.052** | **>0.001** | **5.850** | **>0.001** |
| *R. - Heleioporus* | **3.850** | **>0.001** | 0.196 | >0.001 | 0.343 | >0.001 | 0.237 | >0.001 | 0.231 | >0.001 | 0.990 | 0.811 | **1.365** | **>0.001** |
| *R. - Lechriodus* | **490.322** | **>0.001** | 1.954 | 0.546 | 0.449 | 0.157 | 1.293 | 0.906 | 1.055 | 0.888 | **9.163** | **>0.001** | **1.379** | **>0.001** |
| *R. - Limnodynastes* | **2.866** | **>0.001** | 0.159 | >0.001 | 0.302 | >0.001 | 0.251 | >0.001 | 0.174 | >0.001 | **1.203** | **>0.001** | 0.331 | >0.001 |
| *R. - Metacrinia* | **209.100** | **>0.001** | 0.549 | 0.277 | 2.195 | 0.464 | 0.807 | 0.611 | 1.566 | 0.726 | **15.776** | **>0.001** | **27.774** | **>0.001** |
| *R. - Mixophyes* | **5.657** | **>0.001** | 0.272 | >0.001 | 0.496 | 0.018 | 0.442 | 0.006 | 0.571 | 0.058 | **8.902** | **>0.001** | **1.401** | **>0.001** |
| *R. - Myobatrachus* | **8.994** | **0.021** | 0.275 | 0.013 | 0.715 | 0.480 | 0.990 | 0.842 | 0.343 | 0.043 | **9.701** | **>0.001** | **2.879** | **>0.001** |
| *R. - Neobatrachus* | **8.139** | **>0.001** | 0.331 | >0.001 | 0.449 | 0.005 | 0.318 | >0.001 | 0.408 | 0.002 | **1.092** | **0.007** | **2.165** | **>0.001** |
| *R. - Notaden* | **21.055** | **>0.001** | 0.328 | 0.001 | 0.582 | 0.125 | 0.718 | 0.341 | 1.027 | 0.993 | **1.738** | **>0.001** | 0.208 | >0.001 |
| *R. - Paracrinia* | **31.795** | **0.004** | 0.741 | 0.528 | 0.685 | 0.455 | 1.101 | 0.932 | 0.265 | 0.018 | **9.199** | **>0.001** | **1.442** | **>0.001** |
| *R. - Philoria* | **11.036** | **>0.001** | 0.726 | 0.307 | 0.575 | 0.080 | 0.535 | 0.047 | 0.297 | >0.001 | **5.847** | **>0.001** | 1.146 | 0.089 |
| *R. - Platyplectrum* | **42.456** | **>0.001** | 0.561 | 0.187 | 0.673 | 0.354 | 0.431 | 0.055 | 0.344 | 0.014 | 1.010 | 0.837 | 0.313 | >0.001 |
| *R. - Pseudophryne* | **62.109** | **>0.001** | 0.280 | >0.001 | 0.683 | 0.150 | 0.435 | 0.002 | 0.693 | 0.165 | **1.573** | **>0.001** | 0.523 | >0.001 |
| *R. - Rheobatrachus* | **3.651** | **0.042** | 0.360 | 0.019 | 0.316 | 0.008 | 0.215 | >0.001 | 0.594 | 0.232 | **19.556** | **>0.001** | 0.780 | 0.419 |
| *R. - Spicospina* | **85.036** | **>0.001** | 0.888 | 0.708 | 1.612 | 0.700 | 0.532 | 0.256 | 0.642 | 0.398 | **68.568** | **0.029** | 37.664 | 0.052 |
| *R. - Taudactylus* | **109.047** | **>0.001** | 0.655 | 0.179 | 0.704 | 0.265 | 0.566 | 0.072 | 0.510 | 0.033 | **4.039** | **>0.001** | 0.651 | 0.005 |
| *R. - Uperoleia* | **33.443** | **>0.001** | 0.235 | >0.001 | 0.482 | 0.003 | 0.407 | >0.001 | 0.427 | >0.001 | 0.909 | >0.001 | 0.259 | >0.001 |
