# Supplementary material for: Invasive cane toads are unique in shape but overlap in ecological niche compared to Australian native frogs
Source: Ecol Evol. 2017 Aug 17;7(19):7609–19. doi: 10.1002/ece3.3253 (PMC5632638; doi:10.1002/ece3.3253)
Supplement: Supplementary file 14 [file ECE3-7-7609-s014.docx]

| Table S8. PCA loadings for the environmental dataset. | | | | | |
| --- | --- | --- | --- | --- | --- |
|  |  |  |  |  |  |
| **Axis** | **1** | **2** | **3** | **4** | **5** |
| **Eigenvalues (λ)** | **5.28** | **2.70** | **1.21** | **0.80** | **0.74** |
| **Total variance explained (%)** | **44.00** | **22.53** | **10.06** | **6.69** | **6.16** |
| Annual mean Evaporation | 13.55458 | 2.77823 | 2.08084 | 0.07154 | 6.13442 |
| Precipitation in the driest quarter (Bio17) | 14.22807 | 0.67199 | 1.91810 | 0.41009 | 14.73124 |
| Precipitation - seasonality (Bio15) | 5.77931 | 17.83625 | 5.44484 | 0.05249 | 11.88124 |
| Precipitation in the warmest quarter (Bio18) | 2.06670 | **27.58060** | 2.60953 | 1.08104 | 3.15026 |
| Radiation - seasonality (Bio23) | 3.73493 | **26.45529** | 0.26237 | 0.01980 | 0.66418 |
| Radiation in the warmest quarter (Bio26) | 13.21448 | 6.67878 | 0.08268 | 2.53223 | 1.80336 |
| Temperature in the warmest quarter (Bio10) | 9.99216 | 5.91473 | 2.17120 | 0.07182 | 6.53177 |
| Soil nutrient status | 0.13675 | 0.00105 | **53.40673** | 20.17757 | **24.82375** |
| Annual mean moisture index (Bio28) | **16.60637** | 2.51964 | 0.62267 | 0.65114 | 0.00785 |
| Highest quarter mean moisture index (Bio32) | 7.80625 | 5.92802 | 17.24485 | 0.91931 | 18.36149 |
| Topographic slope (degrees) | 3.28956 | 1.27470 | 11.82740 | **73.21632** | 6.33160 |
| Mean net primary productivity | 9.59083 | 2.36072 | 2.32877 | 0.79665 | 5.57883 |
