# Supplementary material for: Invasive cane toads are unique in shape but overlap in ecological niche compared to Australian native frogs
Source: Ecol Evol. 2017 Aug 17;7(19):7609–19. doi: 10.1002/ece3.3253 (PMC5632638; doi:10.1002/ece3.3253)
Supplement: Supplementary file 15 [file ECE3-7-7609-s015.docx]

| Table S9. Bartlett's test of homogeneity of variances per clade for each environmental variable | | | |
| --- | --- | --- | --- |
| **Variable** | **Bartlett's K-squared** | **d.f.** | **p-value** |
| Annual mean Evaporation | 25422.29000 | 45 | < 0.001 |
| Precipitation in the driest quarter (Bio17) | 14163.22000 | 45 | < 0.001 |
| Precipitation - seasonality (Bio15) | 35619.39000 | 45 | < 0.001 |
| Precipitation in the warmest quarter (Bio18) | 12926.29000 | 45 | < 0.001 |
| Radiation - seasonality (Bio23) | 21281.4 | 45 | < 0.001 |
| Radiation in the warmest quarter (Bio26) | 17402.2 | 45 | < 0.001 |
| Temperature in the warmest quarter (Bio10) | 18476.32 | 45 | < 0.001 |
| Soil nutrient status | 1940.108 | 45 | < 0.001 |
| Annual mean moisture index (Bio28) | 12595.33 | 45 | < 0.001 |
| Highest quarter mean moisture index (Bio32) | >10^6 | 45 | < 0.001 |
| Topographic slope (degrees) | 13636.6 | 45 | < 0.001 |
| Mean net primary productivity | 9506.542 | 45 | < 0.001 |
